# The Genome of the Moderate Halophile *Amycolicicoccus subflavus* DQS3-9A1^T^ Reveals Four Alkane Hydroxylation Systems and Provides Some Clues on the Genetic Basis for Its Adaptation to a Petroleum Environment

**DOI:** 10.1371/journal.pone.0070986

**Published:** 2013-08-14

**Authors:** Yong Nie, Hui Fang, Yan Li, Chang-Qiao Chi, Yue-Qin Tang, Xiao-Lei Wu

**Affiliations:** 1 Department of Energy and Resources Engineering, College of Engineering, Peking University, Beijing, P. R. China; 2 Institute of Engineering (Baotou), College of Engineering, Peking University, Baotou, China; Belgian Nuclear Research Centre SCK/CEN, Belgium

## Abstract

The moderate halophile *Amycolicicoccus subflavus* DQS3-9A1^T^ is the type strain of a novel species in the recently described novel genus *Amycolicicoccus*, which was isolated from oil mud precipitated from oil produced water. The complete genome of *A. subflavus* DQS3-9A1^T^ has been sequenced and is characteristic of harboring the genes for adaption to the harsh petroleum environment with salinity, high osmotic pressure, and poor nutrient levels. Firstly, it characteristically contains four types of alkane hydroxylases, including the integral-membrane non-heme iron monooxygenase (*AlkB*) and cytochrome P450 CYP153, a long-chain *alkane* monooxygenase (LadA) and propane monooxygenase. It also accommodates complete pathways for the response to osmotic pressure. Physiological tests proved that the strain could grow on *n*-alkanes ranging from C10 to C36 and propane as the sole carbon sources, with the differential induction of four kinds of alkane hydroxylase coding genes. In addition, the strain could grow in 1–12% NaCl with the putative genes responsible for osmotic stresses induced as expected. These results reveal the effective adaptation of the strain DQS3-9A1^T^ to harsh oil environment and provide a genome platform to investigate the global regulation of different alkane metabolisms in bacteria that are crucially important for petroleum degradation. To our knowledge, this is the first report to describe the co-existence of such four types of alkane hydroxylases in a bacterial strain.

## Introduction

Microbial enhanced oil recovery (MEOR) is one of the tertiary methods purported to increase oil recovery. Degradation of hydrocarbons such as *n*-alkanes is important for the successful application of MEOR, as well as bioremediation of environments polluted by petroleum [1], [2]. Microorganisms can produce a number of metabolites, such as acids, solvents, biosurfactants, and biopolymers from hydrocarbon metabolisms, which can increase the fluidity and decrease the viscosity of the oil. Microorganisms have evolved different mechanisms to hydroxylate *n*-alkanes and to adapt to the harsh petroleum environments. For example, several types of alkane hydroxylases have been characterized to hydroxylate *n*-alkanes to alcohols, which are further oxidized to the corresponding aldehydes and fatty acids and finally converted to acetyl-CoA via the β-oxidation pathway. The integral-membrane non-heme di-iron monooxygenase (AlkB) [3] and the cytochrome P450 CYP153 family alkane hydroxylases [4] catalyze the hydroxylation of medium-chain-length alkanes, with the carbon atoms ranging from C8 to C16 [5]. The soluble non-heme di-iron monooxygenases (sMMO) and membrane-bound particulate copper-containing enzymes (pMMO) are responsible for the metabolism of short gaseous alkanes [6], [7]. The flavin-binding monooxygenase (AlmA) and a long chain *alkane* monooxygenase (LadA) have been reported to be involved in long-chain alkane (>C18) metabolism [8], [9]. In addition, a novel gene encoding AlkB-rubredoxin fusion protein (AlkW) was recently identified, and it is responsible for the degradation of alkanes ranging from C14 to C36 [10]. These hydroxylases always have the specific substrates spectrum with a narrow range of alkane chain length. However, an increasing number of bacteria have been isolated with the ability to degrade a wide range of hydrocarbons [11], including *Dietzia* strains [12]. This leads to the question of how a single bacterium can degrade alkanes with a wide range of chain lengths. Although recent studies have revealed that AlkB-like and CYP 153-like alkane hydroxylases are sometimes present in the same host bacterial strain [12]–[14], it is still unclear whether the degradation of a wide range of *n*-alkanes is attributable to some unknown hydroxylation pathways or to the co-existence of multiple types of hydroxylases in the same strain.

Recently, we isolated a moderate halophile *Amycolicicoccus subflavus* DQS3-9A1^T^ with the ability to degrade crude oil and grow in 1–12% (wt/vol) NaCl [15]. The genome sequence suggested that the strain harbored *alkB* homologous genes and a complete *n*-alkane metabolism pathway [16]. Here, we report that the strain harbors multiple types of alkane hydroxylases, which allow the strain to hydroxylate and grow on *n*-alkanes with different chain lengths. In addition, the genes required for living in environments with different salinities have been analyzed. Further transcriptional analysis suggested the possible functions of these genes. To our knowledge, this is the first report that a halophilic bacterial strain can harbor most of the known hydroxylase gene homologs that allow the strain to grow on a wide range of *n*-alkanes.

## Materials and Methods

### Isolation and Genomic Analysis of *A. subflavus* DQS3-9A1^T^

The strain *A. subflavus* DQS3-9A1^T^ was isolated from oil “mud,” which was sedimented from oil produced water at Daqing Oilfield, China [15]. The concentration of total dissolved salts in the oil produced water was around 8,000 mg/liter, and the temperatures of the reservoir working strata and the produced water were both 45°C. Sequencing of the complete genome (CP002786 [chromosome], CP002787 [pAS9A-1], and CP002788 [pAS9A-2]) of *A. subflavus* DQS3-9A1^T^ was performed with a combined strategy of 454 sequencing [17] and Solexa paired-end sequencing technology [18], as described previously [16].

Protein coding genes were predicted using Glimmer 3.0 [19], and Genomic islands (GIs) were analyzed using IslandViewer (http://www.pathogenomics.sfu.ca/islandviewer) [20]. The genome sequence was also submitted to the Integrated Microbial Genomes (IMG) database (http://img.jgi.doe.gov) of the Joint Genome Institute for detailed analysis and genome comparison [21]. A one-sample *t*-test was used to evaluate the statistically significant differences of the function profiles based on cluster of orthologous groups (COG) between *A. subflavus* DQS3-9A1^T^ and genomes deposited in the IMG bacteria genome database. Briefly, the data about the abundance of a COG category distributed in each genome were transformed into normal distribution data using Box-cox transformation [22], and then the one-sample *t*-test was performed using the SPSS program (SPSS Inc., IL, US). Genes that could be functionally assigned according to the KEGG Orthology classification system (File S2) were used for constructing the metabolic pathways using iPATH (http://pathways.embl.de/iPath2.cgi) [23].

### Growth of the Strain on Different *n*-alkanes and in Different NaCl Concentrations

To examine growth on *n*-alkanes, the strain DQS3-9A1^T^ was first grown in lysogeny broth (LB) at 30°C till the middle exponential phase. The cells were then collected by centrifugation (1,000×*g* for 5 min, 4°C), washed 3 times with sterile phosphate-buffered saline, and finally suspended in sterile minimal salt medium (MSM; 5 g NaCl, 1 g NH_4_H_2_PO_4_, 1 g (NH_4_)_2_SO_4_, 1 g K_2_HPO_4_, 0.2 g MgSO_4_, and 3 g KNO_3_ per liter of deionized water, pH = 7.2) to make the inoculum cell suspension. For growth tests, the cell suspension was inoculated into 100 mL of MSM in 300-mL flasks (with the final cell concentration in the culture being OD_600_ = 0.05) containing 0.1% (vol/vol) MT microelements (MT stock contains 2.78 g FeSO_4_·7 H_2_0, 1.98 g MnCl_2_·4 H_2_0, 2.81 g CoS0_4_·7 H_2_0, 1.47 g CaCl_2_·2 H_2_0, 0.17 g CuCl_2_·2 H_2_0, and 0.29 g ZnSO_4_·7 H_2_0 in 1 N HCl per liter of deionized water), supplemented with 0.2% liquid *n*-alkanes (C10, C14, C16 and C18) (vol/vol) or 0.1% solid *n*-alkanes (C24, C28, C32, and C36) (wt/vol) as the carbon sources. To examine the growth on gas alkanes (ethane, propane, butane and pentane), the cell suspension was inoculated into 40 mL of the above MSM containing MT microelements in 100-mL gas-tight serum bottles with 60-mL headspace of air. Then 20 mL of gas alkanes were pressurized into each bottle using syringe. Cultures with 1% (wt/vol) glucose as the sole carbon source were used for comparison. In addition, cultures containing cells without carbon source were used as controls for calculating the background growth baseline. Cell growth was detected by the increase of OD_600_ in the culture with time. Six duplicates (parallel flasks) of all cultures were made and incubated in the dark at 30°C while shaking at 150 rpm. To examine the transcriptional levels of putative genes for alkane degradation, three parallel flasks of each culture in the middle exponential phase were sampled for RNA extraction and reverse transcription PCR. Cells grown in 1% glucose were collected as the control for transcriptional analysis. For the *n*-alkane degradation test, the cell suspension was inoculated into 100 mL of the above MSM containing MT microelements, supplemented with C14, C16, C20, C24, C28, C32, and C36 *n*-alkanes as the sole carbon source. Cultures containing hydrocarbons without cells were used as controls for calculating the background evaporation of the *n*-alkanes. At 8 days after incubation, residual *n*-alkanes in the culture were extracted and detected, as described previously [12].

To test growth with different NaCl concentrations, cells were grown in modified artificial seawater (ASW) medium [15] (5 g peptone, 1 g yeast extract, 4 g Na_2_SO_4_, 0.68 g KCl, 0.1 g KBr, 0.025 g H_3_BO_3_, 5.4 g MgCl_2_·H_2_O, 1.5 g CaCl_2_·2H_2_O, 0.024 g SrCl_2_·6H_2_O, 0.2 g NaHCO_3_, 0.04 g Na_2_HPO_4_, 0.5 g NH_4_Cl, and 0.002 g NaF per liter of deionized water, pH 8.0), with NaCl added at the indicated concentration when necessary. The cell growth was determined by detecting OD_600_ with time. To examine the transcriptional levels of putative genes for osmosensing and responding, cells were grown in the modified ASW medium till the middle exponential phase, followed by the addition of NaCl into the medium at a final concentration of 4% NaCl. Cells were then collected at 15 min, 30 min, 60 min, and 24 h after NaCl was added. Cells harvested before NaCl was added were used as the control for transcriptional analysis.

### Real-time Reverse Transcription PCR

Immediately after the samples for real-time reverse transcription PCR were obtained from the cultures, ice-cold stop solution (10% water-saturated phenol in ethanol, pH 5.0) was added at 10∶1 (vol:vol) ratio. Samples were then maintained on ice for 10 min before being collected using centrifugation (6,000×*g* for 5 min, 4°C). Total RNA from the above samples was extracted using TRIzol Reagent (Invitrogen), treated with DNase I, and purified according to the manufacturer’s instructions. Reverse transcription was performed using 0.5 µg of total RNA with random primers and the ReverTra Ace reverse transcription kit (ToYoBo). Specific cDNA was then quantified using real-time PCR (Bio-Rad CFX real-time PCR system; Bio-Rad) with the SYBR Green Ex Taq Kit II (TaKaRa). The specific gene primer pairs are shown in Table S1 in File S1. The gene expression level was normalized against the 16S rRNA gene and calculated using the ΔΔC_t_ method [24]. Cells grown in MSM with glucose as the sole carbon source and cells grown in ASW before NaCl was added were used as the control for transcriptional analysis of cells grown in *n*-alkanes and cells responding to osmotic stress, respectively. All the experiments were performed in triplicate.

## Results

### Characteristic Genome Features of *Amycolicicoccus subflavus* DQS3-9A1^T^

**Figure 1:**
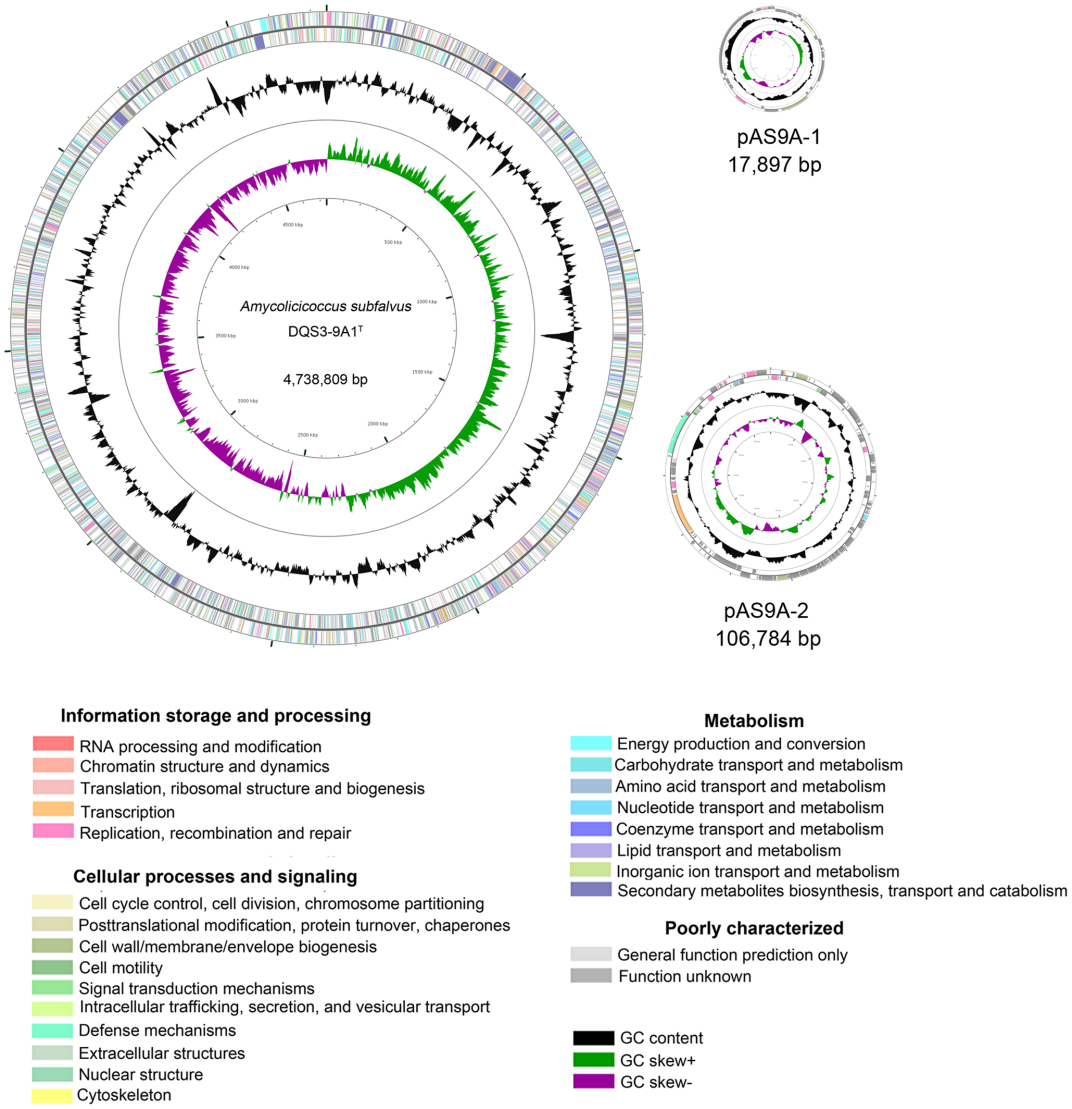
The circular chromosome of *Amycolicicoccus subflavus* DQS3-9A1^T^. The scale on the outside indicates the size. Position 1 of the chromosome was assigned to the first nucleotide of the *dnaA* gene. Rings 1 and 2 (from the outside in) indicate the genes in the forward and reverse strands, respectively, and the colors of the genes indicate the COG categories. Rings 3 and 4 indicate the G+C content and GC skew [(C−G)/(C+G)], respectively. The circular genome map was generated using CGview [49].

**Figure 2:**
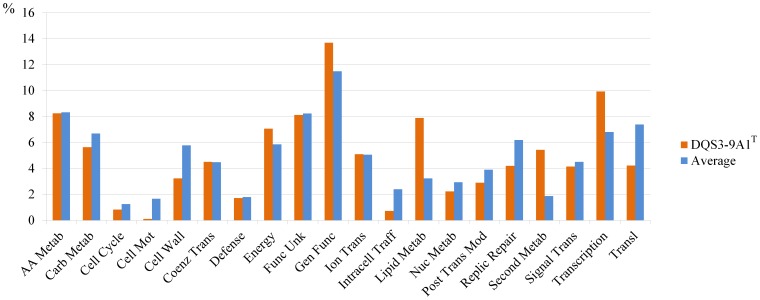
The comparison of gene abundance in COG categories between DQS3-9A1^T^ and other bacterial genomes in the IMG bacterial genome collection.

The complete genome of *A. subflavus* DQS3-9A1^T^ comprises a circular chromosome (4,738,809 bp) and two plasmids (pAS9A-1 [17,897 bp] and pAS9A-2 [106,784 bp]) (Figure 1) that contain 4,557, 27, and 121 predicted protein-coding genes, respectively (15). Of the total 4,705 predicted protein-coding genes, 3,274 could be assigned to COGs (Table 1) and distributed into 22 different categories (Figure 2).

**Table 1:** The key features of the complete genome of *Amycolicicoccus subfalvus* DQS3-9A1^T^.

Feature	Chromosome	pAS9A-1	pAS9A-2
**Size (bp)**	4,738,809	17,897	106,784
**G+C content (%)**	62.24	63.87	61.86
**protein coding genes**	4,557	27	121
**Coding density**	91.29%	80.4%	84.02%
**Avg of CDS length** **(bp)**	949	533	741
**Genes with COGs**	3,235	5	34
**No. of tRNA genes**	45	NA	NA
**No. of rRNA operons**	3	NA	NA
**No. of misc RNA genes**	3	NA	NA

Comparisons of each COG category with other 2,633 genomes in the IMG database revealed that the genome of DQS3-9A1^T^ was significantly (P<0.001) more abundant in the genes responsible for energy production and conversion (C); lipid transport and metabolism (I); secondary metabolite biosynthesis, transport, and catabolism (Q); and transcription (K) (Figure 2 and Table S2 in File S1). In contrast, although complete Embden-Meyerhof (EM) and Entner-Doudoroff (ED) glycolysis pathways were identified, the abundance of genes responsible for carbohydrate transport and metabolism (G) was significantly (P<0.001) lower, including but not limited to the genes in cell cycle control, cell division and chromosome partitioning (D), and cell motility (N) (Figure 2 and Table S2 in File S1).

The DQS3-9A1^T^ genome is also characteristic, with its low content of potential mobile genetic elements. Six and 2 GIs were predicted using the IslandPath - DIMOB and SGI-HMM [25] methods, respectively (Figure S1, Table S3, and Table S4 in File S1). A comparison between DQS3-9A1^T^ and 118 bacteria selected to represent the gene transfer of all bacterial genomes in the IMG collection [25] revealed significantly less gene transfer potential in strain DQS3-9A1^T^ (Table S5 in File S1). In addition, among the 111 predicted genes identified in GIs, many were associated with osmotic stress response, energy metabolism, and hydrocarbon degradation (Table S3 in File S1).

**Figure 3:**
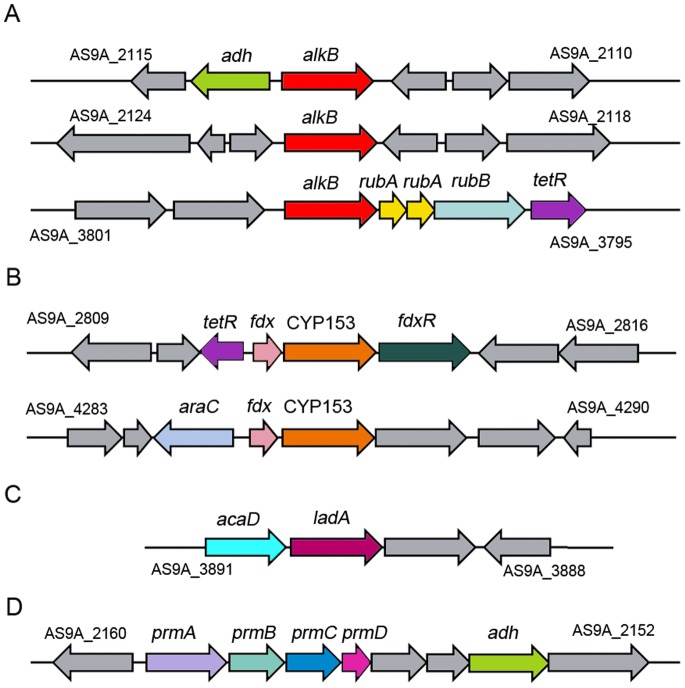
The gene clusters involved in alkane degradation. A: AlkB. B: CYP153. C: LadA. D: propane monooxygenase. Displayed genes and encoded enzymes: *adh*, alcohol dehydrogenase; *rubA*, rubredoxin; *rubB*, rubredoxin reductase; *tetR*, transcriptional regulator, TetR family; *araC*, transcriptional regulator, AraC family; *fdx*, ferredoxin; *fdxR*, ferredoxin reductase; *prmA*, propane monooxygenase large subunit; *prmB*, propane monooxygenase reductase; *prmC*, propane monooxygenase small subunit; *prmD*, coupling protein.

Most strikingly, four types of alkane hydroxylase coding genes were identified in the genome (Figure 3 and Table S6 in File S1). The first type was the AlkB-like alkane hydroxylases, containing 3 homologous genes (AS9A_2113, AS9A_2121, and AS9A_3799) (Figure 3A) with 70%, 61%, and 64% amino acid identity to AlkB1 and 63%, 68%, and 69% amino acid identity to AlkB2 from *Rhodococcus* sp. strain Q15. AlkB1 and AlkB2 in the strain Q15 were responsible for the initial oxidation of alkanes with chain lengths longer than C16 and ranging from C10 to C16, respectively [26]. Secondly, two genes coding for CYP153 alkane hydroxylases (AS9A_2813 and AS9A_4287) (Figure 3B) were identified. The CYP153 gene AS9A_2813 shared 70% amino acid identity with that from *Gordonia neofelifaecis* NRRL B-59395 and AS9A_4287 shared 96% amino acid identity with that of *Dietzia cinnamea* P4, respectively. It was notable that gene AS9A_4287 coding for CYP153 was in a predicted GI (2,023,735–2,067,073 bp). The third type of hydroxylase found was a LadA homolog coding gene (AS9A_3890) (Figure 3C) with 52% amino acid identity with LadA from *Geobacillus thermodenitrificans* NG80-2 that was confirmed to be an extracellular protein able to convert *n*-alkanes, ranging from C16 to C32, into alcohols [8]. Moreover, a gene cluster *prmDCBA* (AS9A_2156–AS9A_2159) encoding multiple components of propane monooxygenase was also found in the genome of DQS3-9A1^T^ (Figure 3D), with 90%, 88%, 88%, and 97% identity with the homologous genes from *Rhodococcus jostii* RHA1 [27], which were annotated as monooxygenase component, monooxygenase hydroxylase, phenol hydroxylase, and propane monooxygenase hydroxylase large subunit, respectively. In addition, genes for alcohol and aldehyde dehydrogenases, as well as those required for fatty acid metabolism, were identified in the genome. The presence of these genes would ensure the complete degradation of *n*-alkanes after they were hydroxylated [28].

**Figure 4:**
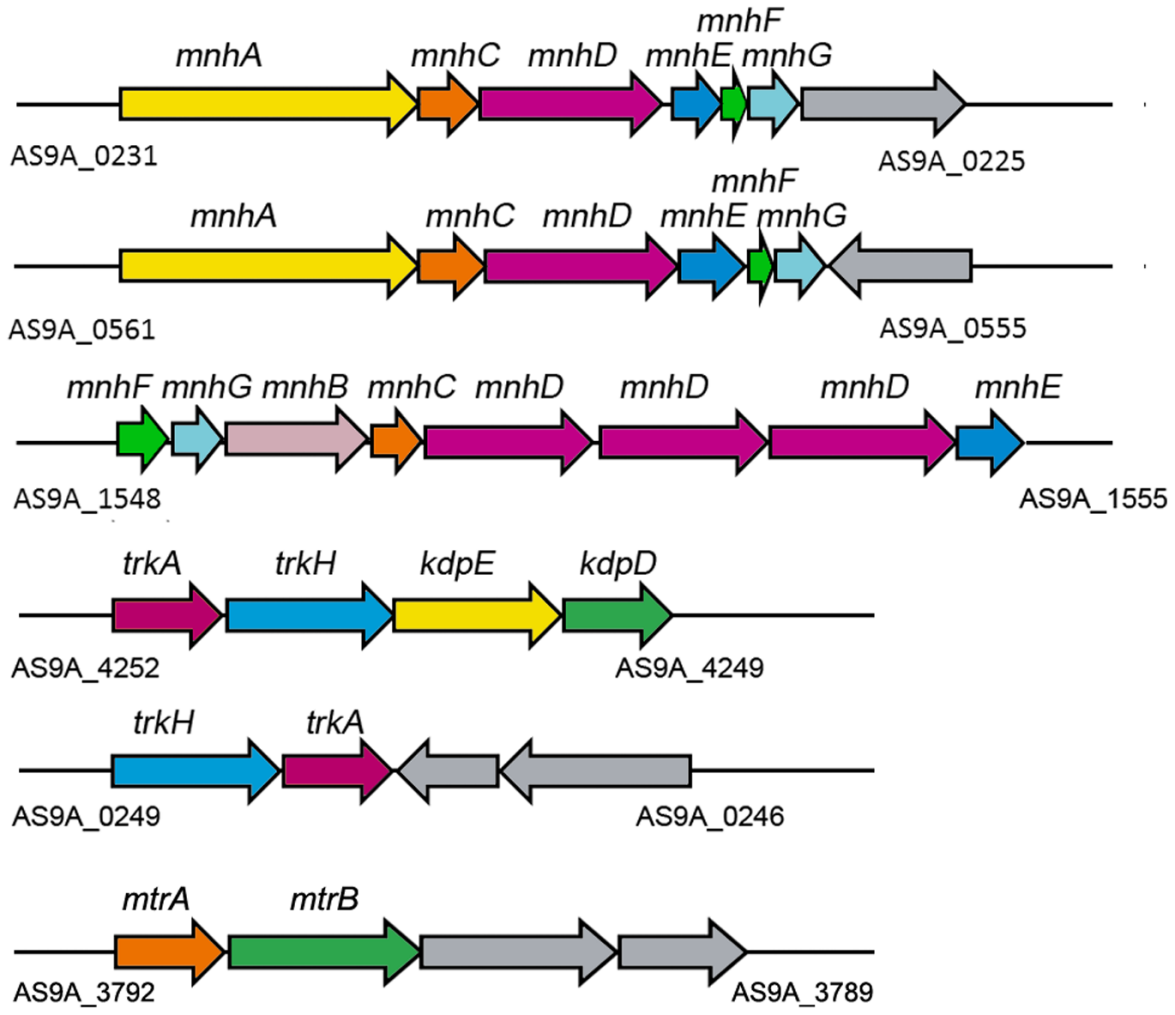
The gene clusters involved in the response to osmotic stress. Displayed genes and encoded transporters: *mnh*, multi-subunit Na^+^/H^+^ antiporter; *nha*, Na^+^/H^+^ antiporter; *trk*, Trk potassium uptake system; *kdp*, KdpD/KdpE two component system; *mtr*, MtrA/MtrB two component system.

Bacteria are frequently exposed to osmotic stresses. In defense, bacteria may accumulate compatible solutes like amino acids and their derivatives, oligosaccharides, and glycosides in their cytoplasm. A number of osmoregulated receptors and transport systems have been investigated. These include KdpD/KdpE and EnvZ/OmpR two component systems (TCSs) from *Escherichia coli*, MtrA/MtrB TCS from *Corynebacterium glutamicum* for osmosensing, Trk-type K^+^ transport systems [29] for K^+^ accumulation, and glycine betaine transporter (BetP) for the uptake of compatible solutes [30]. The synergistic effects of TCSs and corresponding transporters might accompany a short-term response by changing the membrane permeability, and a long-term response through the stable expression of osmoresponsive genes [31]. Although the strain DQS3-9A1^T^ was isolated from oil “mud” sedimented by oil produced water with the concentration of total dissolved solids of around 8,000 mg/L, it could grow in 1–12% NaCl. A number of genes for osmosensing and regulation genes were found in the DQS3-9A1^T^ genome (Table S6, Table S7, and Table S8 in File S1). These included genes encoding up to 16 complete TCSs, among which 10 TCSs could be functionally assigned according to the KEGG Orthology (KO) classification system (Table S6 in File S1). Of those 10 TCSs, four belonged to the OmpR family, five to the NarL family, and one to the CitB family. For example, MtrA/MtrB (AS9A_3792 and AS9A_3791) and KdpD/KdpE (AS9A_4249 and AS9A_4250) TCSs belong to the OmpR family that has genes responsible for detecting hyperosmotic stress and regulating the expression of genes in cell wall biosynthesis and for the accumulation of compatible solutes [32]. In *Corynebacterium glutamicum*, the MtrA/MtrB TCS was found to regulate the expression of BetP, which was responsible for the accumulation of compatible solutes [33]. KdpD/KdpE could also activate the expression of *kdp* operon, which encoded the high-affinity K^+^ uptake system (Kdp), in response to K^+^ limitation or salt stress [34]. Moreover, genes coding for transport systems, such as Na^+^/H^+^ antiporters (Nha) [35], Trk-type K^+^ transport systems [29] and multi-subunit Na^+^/H^+^ antiporters (Mnh) [36], were found to resist the hyperosmotic environment (Figure 4 and Table S6 in File S1).

### Growth of the DQS3-9A1^T^ Strain on *n*-alkanes and Transcriptional Analysis of Genes Putatively Involved in *n*-alkane Degradation

**Figure 5:**
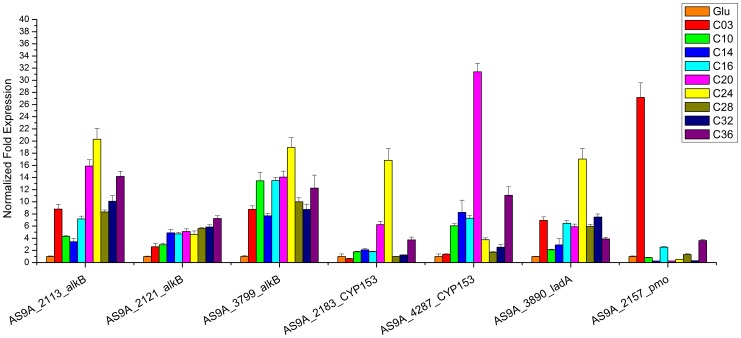
Real-time reverse transcription PCR analysis of the induction of putative genes coding for alkane hydroxylases in cells grown on *n*-alkanes. The *alkB* homologous genes (AS9A_2113, AS9A_2121, and AS9A_3799) were upregulated by C10–C36 *n*-alkanes and propane (C3). The transcription of the CYP153 homologous gene AS9A_2813 was mostly induced by C20 and C24, and another CYP153 coding gene AS9A_4287 was induced by C10 to C20. The transcriptional level of putative *ladA* (AS9A_3890) was induced from C16 to C36. Putative propane oxidation gene (*prmC*) AS9A_2157 was significantly upregulated with propane as the sole carbon source and only slightly induced by C16 and C36. (All data are the mean±SD).

The DQS3-9A1^T^ strain grew with C10–C36 *n*-alkanes and propane as the sole carbon sources (Figure S2 in File S1), leading to the degradation of *n*-alkanes (Figure S3 in File S1). Among the *n*-alkanes, C16 and C18 led to the most rapid and highest growth, while C3 and C10 led to the lowest growth. A moderate growth of the cells was detected in cases of C14 and C20 to C36. Along with the growth of the cells on different *n*-alkanes, the transcriptional levels of the putative alkane hydroxylase genes were detected using quantitative real-time reverse transcription PCR (Figure 5). The transcriptional levels of *alkB* homologous genes (AS9A_2113, AS9A_2121, and AS9A_3799) were upregulated by C10–C36 *n*-alkanes and propane (C3), with the highest expression level detected at C16–C24. The transcription of the CYP153 homologous gene AS9A_2813 was mostly induced by C20 and C24 but not by C10 to C16, and it was distinct from another CYP153 coding gene AS9A_4287, which was induced by C10 to C20. The results suggested the different roles of these two CYP153 genes in the degradation of *n*-alkanes. The transcriptional level of putative *ladA* (AS9A_3890) was induced from C16 to C36, with the highest expression level at C24; this indicated its potential role in the degradation of long-chain-length *n*-alkanes. One of the subunits of the putative propane oxidation gene (*prmC*) AS9A_2157 was significantly upregulated with propane as the sole carbon source and only slightly induced by C16 and C36. The transcriptional profiles of these alkane hydroxylase genes suggested a potential “team” of multiple alkane hydroxylases in strain DQS3-9A1^T^ during *n*-alkane degradation, which needs to be further researched for more biochemical evidences.

### Growth of the Strain Under Different Salinity Conditions and Transcription of Different Putative Genes

**Figure 6:**
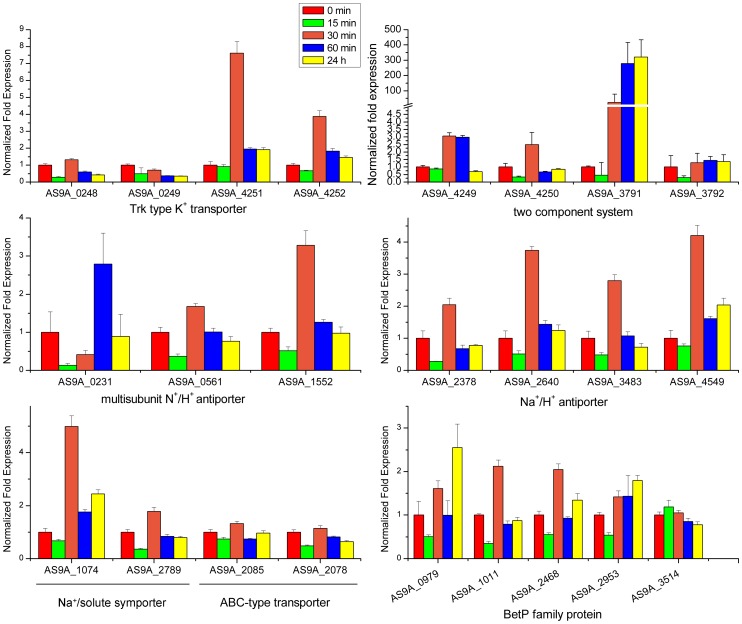
Real-time reverse transcription PCR analysis of the induction of putative genes related to osmotic stress sensing and response in cells exposed to 4% NaCl. Genes coding for KdpD/KdpE TCS, MtrB, Trk-type K^+^ transporter, Nha, Mnh (except the gene AS9A_0231 was induced at 60 min) and Na^+^- and Cl^–^dependent choline cotransporter were induced at 30 min following exposure to additional 4% NaCl. As for the compatible solute transport and synthesis functions, genes (AS9A_0979, AS9A_1011, AS9A_2468, and AS9A_2953) coding for BetP were induced at 30 min after treatment, and the putative *betP* genes AS9A_0978 and AS9A_2953 showed the highest expression level at 24 h after treatment. The ABC-type compatible solute transporter coding genes were not changed. (All data are expressed as mean±SD).

Strain DQS3-9A1^T^ could grow in 1–12% NaCl without significant growth differences between 1% and 4% NaCl (Figure S4 in File S1). The transcriptional levels of the putative genes for osmotic stress sensing and response were detected in DQS3-9A1^T^ after 4% NaCl (final concentration) was added into the culture medium (Figure 6). As for the osmosensing functions, genes coding for KdpD/KdpE TCS (AS9A_4249 and AS9A_4250) were induced at 30 min following exposure to additional 4% NaCl, suggesting their potential function in osmotic sensing and response. After 60 min of exposure, the expression of these two genes decreased to the control level (untreated culture) and was maintained until 24 h, suggesting adaptation of the strain to osmotic stress after 60 min. As for the Na^+^ efflux and K^+^ influx functions, the transcriptional levels of genes coding for Trk-type K^+^ transporter (AS9A_4251 and AS9A_4252), which were flanked by KdpD/KdpE (AS9A_4249 and AS9A_4250) and seemingly grouped together as one operon, were induced after 30 min following exposure and decreased from 60 min to 24 h. In contrast, the expression of another Trk-type K^+^ transporter coding gene (AS9A_0248 and AS9A_0249) did not change with incubation time. Moreover, the transcriptional level of genes coding for Na^+^/K^+^ antiporter (Nha) (AS9A_2378, AS9A_2640, AS9A_3483, and AS9A_4549) were upregulated at 30 min after exposure and were back to the control (untreated culture) level after 60 min. The gene (AS9A_1552) coding for multiple subunits of Na^+^/K^+^ antiporter (Mnh) was also upregulated at 30 min after exposure; however, another gene (AS9A_0231) coding for MnhA was induced at 60 min after exposure. These results suggested that the strain could resist osmotic stress by importing H^+^ and pumping out Na^+^, in addition to K^+^ uptake. Gene encoding MtrB (AS9A_3791), the histidine kinase of the MtrA/MtrB TCS system, was upregulated 30 min after exposure and reached its maximal transcriptional expression level at 60 min and 24 h after exposure. However, expression of the gene encoding MtrA (AS9A_3792), which is the transcriptional regulator of MtrA/MtrB TCS, was not induced after exposure to osmotic stress. As for the compatible solute transport and synthesis functions, genes AS9A_1011 and AS9A_2468 coding for BetP were induced at 30 min after treatment, but the putative *betP* gene AS9A_3514 was not changed. Among these genes, the putative *betP* genes AS9A_0979 and AS9A_2953 showed the highest expression level at 24 h after treatment and were likely to be important for long-term adaptation to osmotic stress. The gene coding for the Na^+^- and Cl^–^dependent choline cotransporter (AS9A_1074) was also upregulated 30 min after treatment, whereas other ABC-type compatible solute transporter encoding genes were not changed.

## Discussion

### Effective Adaptation of DQS3-9A1^T^ to the Oil Reservoir Environment

*A. subflavus* DQS3-9A1^T^ was isolated from oil “mud” that contained sediment from oil produced water. The bacterium has a lower abundance of genes in COG categories of cell duplication (L), intracellular trafficking, secretion, and vesicular transport (U), cell wall/membrane/envelope biogenesis (M), cell motility-related genes (N), as well as genes for carbohydrate transport and metabolism (G), compared with those in bacterial genomes released. The loss of redundant genes in carbohydrate metabolism did not influence the bacterium’s life functions, which was confirmed by the fact that the strain could utilize various carbohydrates, including D-fructose and D-glucose, as the sole carbon source [15]. This characteristic gene composition is very similar with that of *Polymorphum gilvum* SL003B-26A1^T^ [37], which was also isolated from a crude oil-related environment [38].

The genes involved in responding to osmotic stresses provide a clear pathway for osmotic tolerance of DQS3-9A1^T^, including sensing and responding to environmental factors, regulating the expression of target genes, transporting harmful byproducts out of the cells, or producing protective materials. Two temporal gene regulation responses were found during the osmotic stress in strain DQS3-9A1^T^. First, rapid increase of the transcriptional levels of the genes coding for KdpD/KdpE TCS and Trk-like K^+^ transporter suggested that a rapid K^+^ influx by Trk-like K^+^ transporter under the control of KdpD/KdpE TCS and possible accumulation of K^+^ inside the cells was induced to resist the osmotic stress caused by the high concentration of NaCl. Meanwhile, genes encoding Mnh and Nha were also upregulated immediately after osmotic stress, suggesting an immediate efflux of Na^+^ and influx of K^+^ by multiple K^+^ and Na^+^ transporters in the early phase of adaptation.

**Figure 7:**
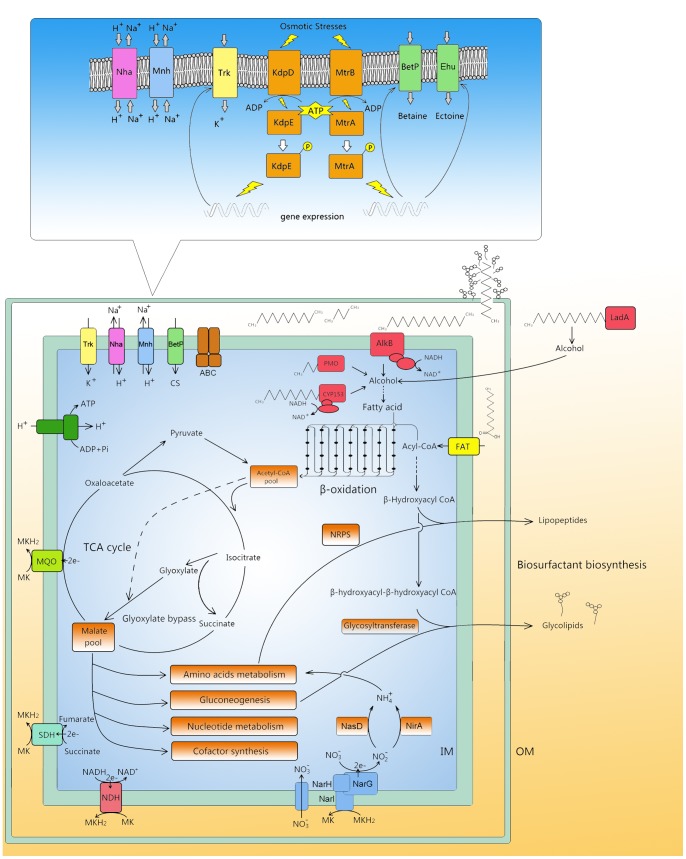
Overall scheme of metabolism and transport in *Amycolicicoccus subflavus* DQS3-9A1^T^ indicating the adaptation of DQS3-9A1^T^ in the crude oil environment. The framed schema shows the model for osmosensing and osmosignaling in DQS3-9A1^T^. IM, inner membrane; OM, outer membrane; NarG, respiratory nitrate reductase catalytic α-subunit; NarH, respiratory nitrate reductase soluble β-subunit; NarI respiratory nitrate membrane biheme *b* quinol-oxidizing γ-subunit (Following the gene designation in *Escherichia coli* [50]); NasD, soluble NADH-nitrite reductase (Following the gene designation in *Bacillus subtilis* [51]); NirA, ferredoxin-nitrite reductase (Following the gene designation in *Synechococcus elongates* [52]) NDH, NADH dehydrogenase; SDH, succinate dehydrogenase; MQO, malate: quinine oxidoreductase; AlkB, integral-membrane non-heme iron monooxygenase; CYP153, cytochrome P450 CYP153 family alkane hydroxylase; PMO, propane monooxygenase; LadA, long-chain length alkane monooxygenase; FAT, fatty acid transporter; NRPS, non-ribosomal peptides synthetase; Trk, *trk* locus-encoded K^+^ uptake system; Nha, Na^+^/H^+^ antiporter; Mnh, multiunit Na^+^/H^+^ antiporter; BetP, betaine and Na^+^ antiporter; Ehu, ectoine/hydroxyectoine ABC transporter.

Second, genes encoding MtrA/MtrB TCS and related to compatible solute transport and synthesis responded later in osmotic adaptation. The putative *betP* genes, AS9A_0978 and AS9A_2953, showed the highest expression levels 24 h after treatment. Moreover, upregulation of the membrane-bound histidine kinase MtrB, which leads to the induction of *betP* [30], suggested the strain’s abilities to produce potential glycine betaine and choline as compatible solutes in the resistance to osmotic stress. It is interesting that although genes encoding MtrA and MtrB were grouped together as one operon, the transcriptional expression of *mtrA* was not induced in osmotic adaptation, compared with the high expression level of *mtrB* 60 min after osmotic stress. This result suggested that *mtrA* and *mtrB* were transcribed by different promoters. A similar case was also observed in *Mycobacterium*, and 2 unique promoters were found in front of the individual genes [39]. In conclusion, genes for Na^+^ efflux and compatible solute transport and biosynthesis were upregulated following exposure of the DQS3-9A1^T^ strain to osmotic stress, e.g., *mnh* operons, Na^+^/H^+^ antiporters, and Trk family K^+^ transporters (Figure 6); this indicated the synergetic work of multiple elements to overcome the hyperosmotic challenge [40] (Figure 7).

The occurrence of horizontal gene transfer (HGT) in DQS3-9A1^T^ appeared to be significantly lower than those in other genomes. This might be attributed to the poor microbial diversities in the oil environment, in comparison with that in other high-diversity environments [41], [42], like soil, which might lead to low opportunities for the exchange of genetic information with the external ecosphere. In a harsh environment that is poor in nutrients, the growth and duplication of cells should be very slow, leading to less activity, even when acquiring foreign genes.

### The DQS3-9A1^T^ Genome Displays Multiple Hydroxylation Systems and Complete Pathways for Hydrocarbon Degradation

For many bacteria living in oil reservoirs, hydrocarbons may be the main carbon resources. The main components of crude oil are aliphatic hydrocarbons (such as *n*-alkanes), which are substrates that are relatively easy to use in comparison with other petroleum components. Alkane degradation is a complex process that includes the synthesis of glycolipids and/or lipopeptides as biosurfactants [43], as well as the degradation of alkane-oriented fatty acids by fatty acid metabolic pathways [28]. It is well documented that *n*-alkane degrading microorganisms have a higher abundance of genes for lipid metabolism [37], as we observed for DQS3-9A1^T^, which had a significantly higher than average abundance of these genes when compared to all known whole genomes in the IMG collection.

One of the most important and outstanding characteristics of the DQS3-9A1^T^ strain is that it contains four types of alkane hydroxylase genes, a unique capacity that, at least to our knowledge, has not been reported before. Among them, the *alkB*-like genes (AS9A_2113, AS9A_2121, and AS9A_3799) could be induced by *n*-alkanes ranging from C10 to C36, suggesting their potential functions in degradation of medium- and long-chain *n*-alkanes. Both CYP153 genes clustered in an operon-like structure, with the difference being that AS9A_4287 was possibly under the regulation of AraC while AS9A_2813 was under the regulation of TetR (Figure 3B). It is known that AraC is a common positive regulator of transcription [44], while TetR is a transcriptional repressor [45]. In gram-negative bacteria, like *Alcanivorax*, CYP153 genes are always regulated by the AraC transcriptional regulator [46], [47] and induced by *n*-alkanes shorter than C18, but not induced by *n*-alkanes longer than C24. Likewise, we found that the CYP153 gene AS9A_4287, which was possibly under the regulation of AraC, was induced by *n*-alkanes ranging from C10 to C20. Furthermore, horizontal gene transfer analysis showed that AS9A_4287 in both the DQS3-9A1^T^ strain and in *Alcanivorax* had the same origin [48]. However, another CYP153 gene AS9A_2813 under the regulation of the TetR-like regulator was mainly induced by C20 and C24. The differential regulation by AraC and TetR regulators might be the source of the different *n*-alkane-degrading capacities of the two genes. Besides the two types of well-documented genes, *alkB* and CYP153, LadA and propane monooxygenase encoding genes were also found. The experiments measuring growth and transcriptional levels of these genes suggested their possible functions with respect to the degradation of longer and gaseous propane *n*-alkanes. Compared to medium-chain-length alkanes, long-chain length alkanes are more persistent in the environments. Moreover, the knowledge about the key enzymes and metabolic regulation in the degradations of long-chain and short-chain alkanes was limited. So more attention should be given to the degradation of long-chain length alkanes and gaseous alkanes.

The high identity of these predicted genes with functionally identified homologs may indicate their functions in the hydroxylation of different chain-length alkanes, which is supported by the results of physiological experiments. However, it is still unclear how these hydroxylases work together to degrade alkanes and the global regulatory mechanism involved. Some recent studies indicate that bacteria may develop strategies to utilize different alkane hydroxylases co-operatively, thus allowing the degradation of a broad range of n-alkanes. This phenomenon has been described for CYP153 and AlkB-like proteins in *Dietzia* sp. DQ12-45-1b [48], as well as CYP153, AlkB, and AlmA in *Alcanivorax dieselolei* B-5 [14] and *Alcanivorax hongdengensis* strain A-11-3 [47]. Further studies, such as gene knockout experiments, are required to verify these functions.

Although genes related to *n*-alkane degradation are abundant in the DQS3-9A1^T^ genome, key genes for the degradation of aromatic compounds were not detected. These include benzoyl CoA synthetase (for benzoate degradation), phenol 2-monooxygenase (for phenol and toluene degradation), and catechol 1,2-dioxygenase and catechol 2,3-dioxygenase (both essential for the metabolism of aromatic compounds). It is interesting to note that *Polymorphum gilvum* SL003B-26A1^T^, isolated from a similar environment as that for DQS3-9A1^T^, displays an opposite gene content in that it actually contains many genes and complete pathways for aromatic compound degradation but relatively few genes for alkane degradation [37].

In conclusion, the genomic, transcriptional, and physiological analyses we describe here show that *A. subfalvus* DQS3-9A1^T^ has an efficient alkane metabolism together with a functioning osmosensing system, allowing it to adapt well to the oil field environment. More specially, the strain DQS3-9A1^T^ harbors propane monooxygenase, CYP153, AlkB and LadA (Table S6 in File S1), as well as genes corresponding to the complete metabolic pathways for fatty acid β-oxidation, amino acid metabolism, nucleotide metabolism and cofactor synthesis, enabling it to use propane and *n*-alkanes ranging from C10 to C36 as the sole carbon source. To our knowledge, this is the first report on the presence of four such enzymes in a single strain. In addition, osmosensing regulators and transporters (Table S6 and Table S7 in File S1), i.e. KdpD/KdpE and MtrA/MtrB two component systems, and Trk K^+^ transporters were detected in the genome strain DQS3-9A1^T^, thus providing a solid genetic basis for strain’s tolerance to osmotic stress. Because it has multiple alkane hydroxylase systems at its disposal combined to hyperosmotic adaptability, the strain DQS3-9A1^T^ showed a strong potential for industrial application in Microbial Enhanced Oil Recovery (MEOR) and bioremediation under saline conditions.

## Supporting Information

File S1 Figure S1, Genomic Island (GI) prediction by different methods. Ring 1 (red) (from outside in) indicates the GIs by multiple methods; ring 2 (blue) indicates the GIs predicted using the IslandPath-DIMOB method; ring 3 (orange) indicates the GIs predicted using the SIGI-HMM method; and the black line plot indicates the G+C content. Figure S2, The growth of *Amycolicicoccus subfalvus* DQS3-9A1^T^ with different *n*-alkanes as the sole carbon source. Figure S3, The degradation ratio of *n*-alkanes by *Amycolicicoccus subfalvus* DQS3-9A1^T^. Figure S4, The growth of *Amycolicicoccus subfalvus* DQS3-9A1^T^ with different concentrations of NaCl. Table S1, Primers used in this study. Table S2, Comparative analysis of COG categories between *Amycolicicoccus subfalvus* DQS3-9A1^T^ and other selected genomes in the IMG bacterial genome database. Table S3, Genomic island prediction by different methods. Table S4, Genes in GIs. Table S5, Comparison of the frequency of gene transfer events between DQS3-9A1^T^ and 118 other bacteria. Table S6, Genes discussed and described in this work. Table S7, Genes involved in TCS. Table S8, Genes in compatible solutes transport and biosynthesis.
(DOCX)

File S2 KO list.KO. The list of KEGG annotation results for construction of metabolic pathways using interactive tools like iPATH.
(KO) Click here for additional data file.
