# Supplementary material for: The Genome of the Moderate Halophile Amycolicicoccus subflavus DQS3-9A1T Reveals Four Alkane Hydroxylation Systems and Provides Some Clues on the Genetic Basis for Its Adaptation to a Petroleum Environment
Source: PLoS One. 2013 Aug 14;8(8):e70986. doi: 10.1371/journal.pone.0070986 (PMC3743902; doi:10.1371/journal.pone.0070986)
Supplement: File S1 — Figure S1, Genomic Island (GI) prediction by different methods. Ring 1 (red) (from outside in) indicates the GIs by multiple methods; ring 2 (blue) indicates the GIs predicted using the IslandPath-DIMOB method; ring 3 (orange) indicates the GIs predicted using the SIGI-HMM method; and the black line plot indicates the G+C content. Figure S2, The growth of Amycolicicoccus subfalvus DQS3-9A1T with different n-alkanes as the sole carbon source. Figure S3, The degradation ratio of n-alkanes by Amycolicicoccus subfalvus DQS3-9A1T. Figure S4, The growth of Amycolicicoccus subfalvus DQS3-9A1T with different concentrations of NaCl. Table S1, Primers used in this study. Table S2, Comparative analysis of COG categories between Amycolicicoccus subfalvus DQS3-9A1T and other selected genomes in the IMG bacterial genome database. Table S3, Genomic island prediction by different methods. Table S4, Genes in GIs. Table S5, Comparison of the frequency of gene transfer events between DQS3-9A1T and 118 other bacteria. Table S6, Genes discussed and described in this work. Table S7, Genes involved in TCS. Table S8, Genes in compatible solutes transport and biosynthesis. (DOCX) [file pone.0070986.s001.docx]

Figure S1 Genomic Islands (GIs) prediction by different methods. Ring 1 (red) (from outside in) indicates the GIs by multiple methods; ring 2 (blue) indicated the GIs predicted by IslandPath-DIMOB method; ring 3 (orange) indicated the GIs predicted by SIGI-HMM method; the black line plot indicates the G+C content.


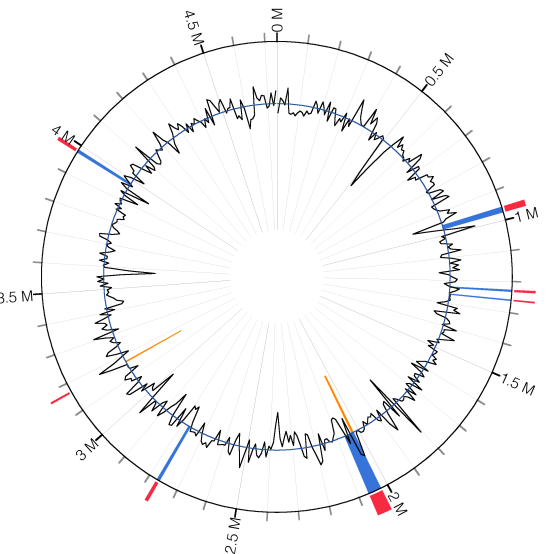


## Figure S2. The growth of *Amycolicicoccus subfalvus* DQS3-9A1^T^ with different *n*-alkanes as the sole carbon source.


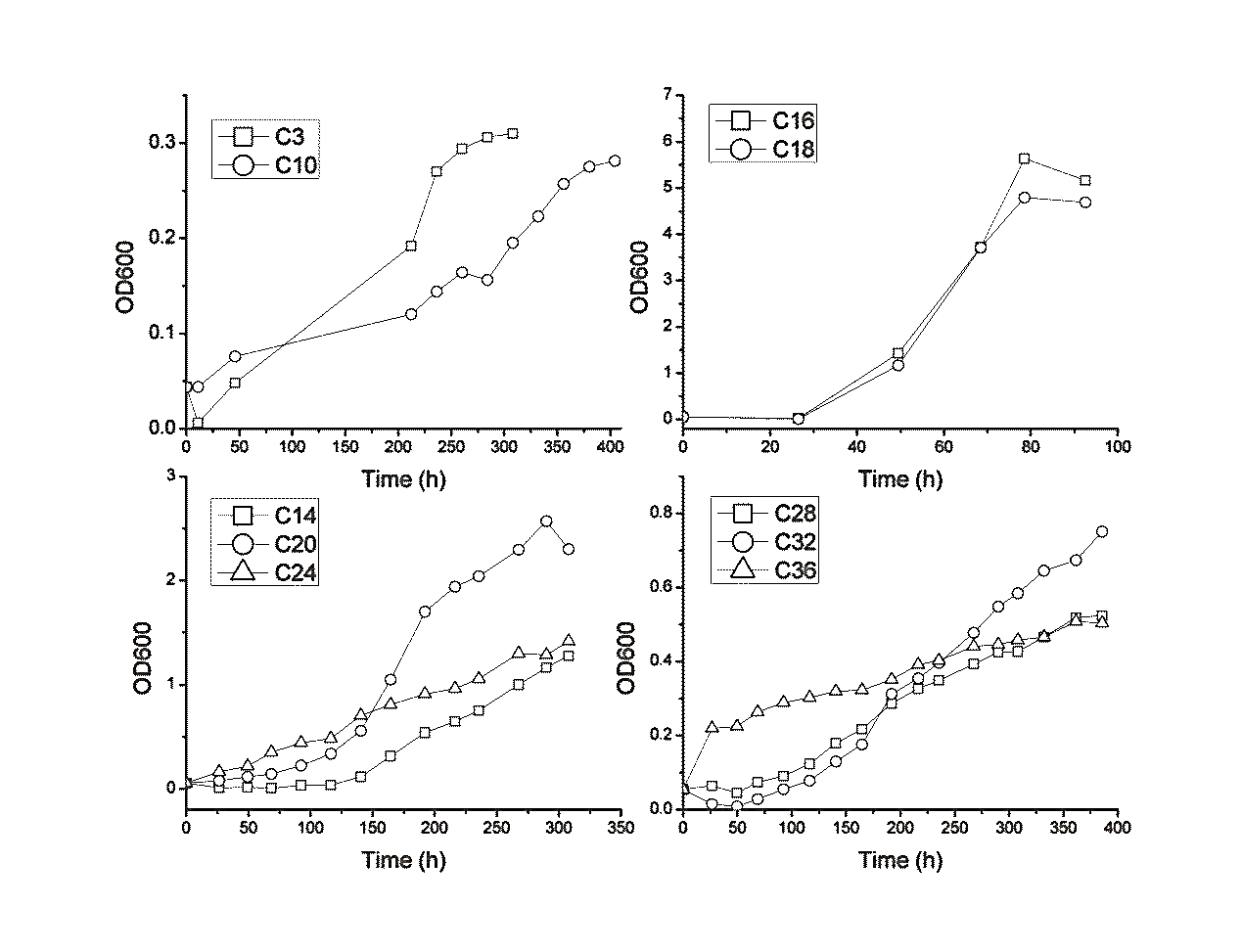


## Figure S3. The degradation ratio of *n*-alkanes by *Amycolicicoccus subfalvus* DQS3-9A1^T^.





## Figure S4. The growth of *Amycolicicoccus subfalvus* DQS3-9A1^T^ with different concentrations of NaCl.


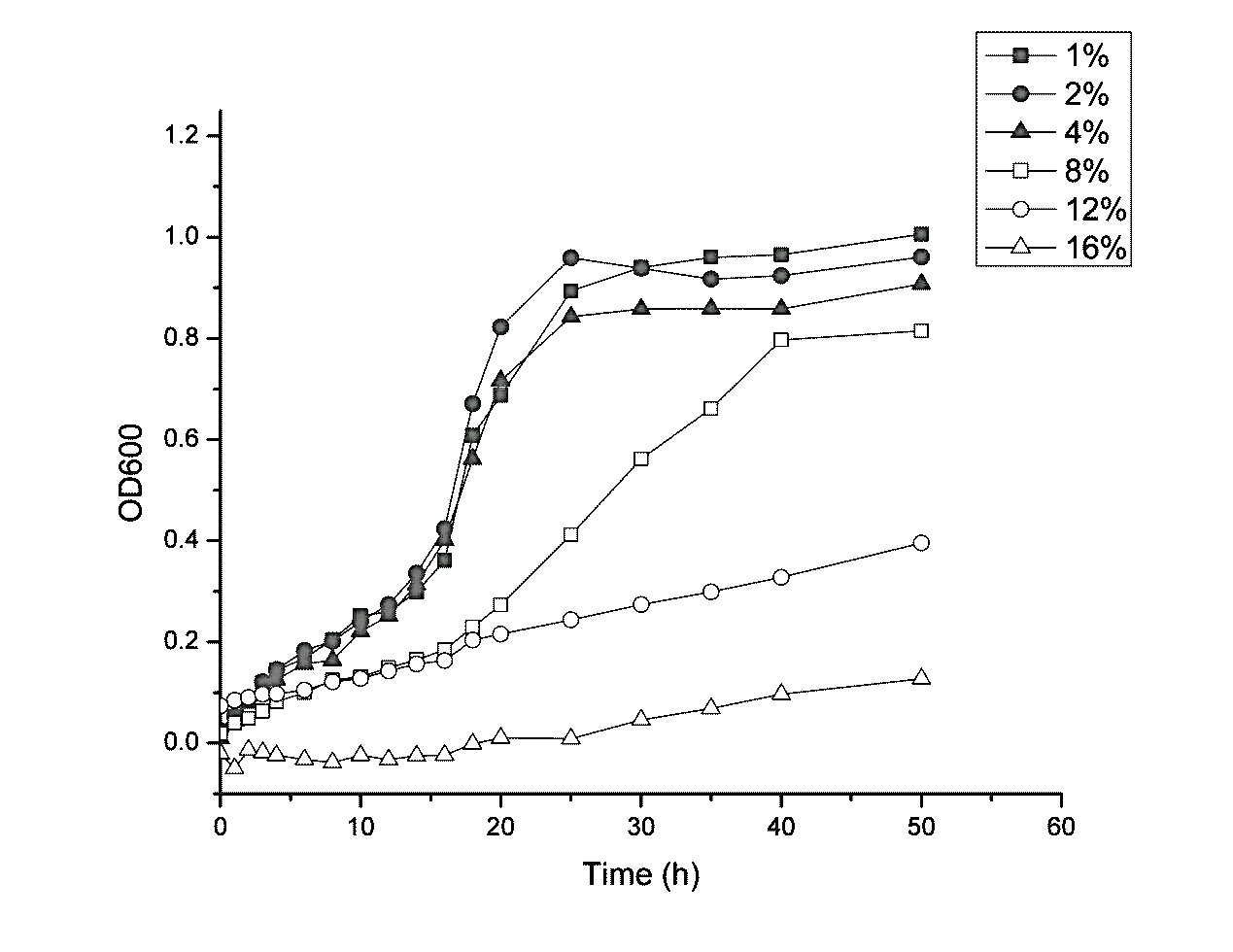


## Table S1 Primers used in this study

| **Name** | **Sequences** |
| --- | --- |
| ABC_AS9A_1074_F | TCGCCCTCATGACGTGGAT |
| ABC_AS9A_1074_R | AGTTCAGTACGGCACCTTCGTCAG |
| ABC_AS9A_2078_F | CCTGGCATTTCCTGTGGGTCTGT |
| ABC_AS9A_2078_R | ATGCCCATCATGACCACGAGCAGA |
| ABC_AS9A_2085_F | TCGAAAACCTGACTGAGGGACCG |
| ABC_AS9A_2085_R | TTGCTGCTGACCGCCTGA |
| ABC_AS9A_2789_F | ATTGTTCCTGGGTTTATCGCGTGT |
| ABC_AS9A_2789_R | AATCGCGCATTGCCGTTTCCAT |
| alkB_AS9A_2113_F | CTCGAAGATCACGCTTGCTCA |
| alkB_AS9A_2113_R | GAATGCCCAGAACGTCTCACC |
| alkB_AS9A_2121_F | ACATTCCCCTGCAGTACGTG |
| alkB_AS9A_2121_R | CCAATGCTCGCCGTAAGCC |
| alkB_AS9A_3799_F | ACATTAAGCGTCGTCGAAAGCCT |
| alkB_AS9A_3799_R | CTTCCTTCTTATGGCCGAGTTCGT |
| betP_AS9A_0979_F | CAGCACTCAGTCACCGCCATCC |
| betP_AS9A_0979_R | CGACGCCCCAAAAGACCAGACCT |
| betP_AS9A_1011_F | TCTGCAAACAGCATCGATTGCCAC |
| betP_AS9A_1011_R | TTGATGGCAGGTCACCCGAA |
| betP_AS9A_2468_F | GCACCCTTTGTCGGGCTC |
| betP_AS9A_2468_R | CCGAGTGCAGTGTTCCCGAAA |
| betP_AS9A_2953_F | TGTTCAACCTTGTGAAAGCCCTC |
| betP_AS9A_2953_R | CTCCAGATCCTGTTGTACGCGGTA |
| betP_AS9A_3514_F | CATCACTCGTCATCCTAGGGCTCA |
| betP_AS9A_3514_R | ACCACCAGCCGACACCGTCA |
| cyp153_AS9A_2183_F | ATCAGCCTGCTCCAATCGCAT |
| cyp153_AS9A_2183_R | TGGTGTCGTTACCGCCTACAAC |
| cyp153_AS9A_4287_F | TACGACAAGAAGGCTCGGACA |
| cyp153_AS9A_4287_R | ACGAGGTTGCCCATGAATTCCA |
| kdpD_AS9A_4249_F | CAGCGACGTCCATTTGACACCC |
| kdpD_AS9A_4249_R | ACCCGCAAGTAGTGAGTTTCGGT |
| kdpE_AS9A_4250_F | GTCAGCCGAGCAAACCAGTC |
| kdpE_AS9A_4250_R | AGCCCAGTCATCCCGAGCAAG |
| ladA_AS9A_3890_F | TGCTCGTCTCCGCTATGGCT |
| ladA_AS9A_3890_R | CGACATTCCATCCGATACGCCCTT |
| mnhA_AS9A_0231_F | CCTCGCGGCACCGTTCGTA |
| mnhA_AS9A_0231_R | GAAACCGACGACGCCGAGCAA |
| mnhA_AS9A_0561_F | GTTTTCACGGTCGCTTACACGTT |
| mnhA_AS9A_0561_R | CCAGTCCACCAGCGGAACTGTCA |
| mnhD_AS9A_1552_F | GCAAGGCTCGGCACCCAC |
| mnhD_AS9A_1552_R | CGACCACACGTTTCAGCTTGCTT |
| MtrA_AS9A_3792_F | ACTGCTCGACCTGATGCTCCC |
| MtrA_AS9A_3792_R | GAGTCCCAGCACAACGTCGACA |
| MtrB_AS9A_3792_F | ACCCCATGCTCCAGCGGTCA |
| MtrB_AS9A_3791_R | TCAATTTGCTCTGCCGCCAGT |
| nha_AS9A_2640_F | AGATTCGTCCAGCGTTGCCGTTC |
| nha_AS9A_2640_R | TGCGACCTGCCAATCGACT |
| nha_AS9A_3483_F | ACGGCGATCGAATCCCTATGGCAA |
| nha_AS9A_3483_R | TCTCTGTCGAGCCATGCCTT |
| nha_AS9A_4549_F | TGCTGATTTCGCGTCCCC |
| nha_AS9A_4549_R | GCAGACCACCCCAAATGACAACA |
| nhaC_AS9A_2378_F | TGCAATCGCTGAACCCGACCA |
| nhaC_AS9A_2378_R | CAAACCGCCCGTAGCAACCTG |
| PMO_AS9A_2157_F | ACGCCCACCCTCGTCGGTA |
| PMO_AS9A_2157_R | TTTATTGCTTTCGCCGTACTCCT |
| trkH_AS9A_4251_F | CGAACTGCGCGGACACCCCA |
| trkH_AS9A_4251_R | TCCCGAACGCTGAAATGACCTC |
| trkA_AS9A_0248_F | TAAGAATGATCGCCAGCAAAGCAC |
| trkA_AS9A_0248_R | GTCAGTTCCCACGGCGACC |
| trkA_AS9A_4252_F | GCGTGCCCAGTCAGAACAACA |
| trkA_AS9A_4252_R | TCGGCAACCTACGTTCGAG |
| trkH_AS9A_0249_F | ATTCCTGCTGTCACTTCCCATCGC |
| trkH_AS9A_0249_R | ACCAGAAGCTGCCCGTATCGAC |

## Table S2. Comparative analysis of COG categories between *Amycolicicoccus subfalvus* DQS3-9A1^T^ and other selected genomes in IMG bacteria genome database.

| COG category | Gene Abundance (%) | | Std. Deviation (%) | Std. Error Mean (%) | *t*-score | *P*-value (2-tailed) |
| --- | --- | --- | --- | --- | --- | --- |
|  | 9A1 | Mean^a^ |  |  |  |  |
| Amino acid transport and metabolism | 8.251423 | 8.23 | 1.97 | 0.038 | 1.898 | 0.058 |
| Carbohydrate transport and metabolism | 5.638903 | 6.69 | 2.75 | 0.053 | 14.834 | 0 |
| Cell cycle control, cell division, chromosome partitioning | 0.827729 | 1.26 | 0.64 | 0.012 | 45.009 | 0 |
| Cell motility | 0.103466 | 1.66 | 1.50 | 0.029 | 65.270 | 0 |
| Cell wall/membrane/envelope biogenesis | 3.233316 | 5.78 | 1.72 | 0.034 | 75.834 | 0 |
| Coenzyme transport and metabolism | 4.500776 | 4.47 | 1.27 | 0..025 | -4.819 | 0 |
| Defense mechanisms | 1.707191 | 1.79 | 0.82 | 0.016 | -10.10 | 0.313 |
| Energy production and conversion | 7.061562 | 5.86 | 1.58 | 0.031 | -39.194 | 0 |
| Function unknown | 8.12209 | 8.23 | 2.03 | 0.04 | 2.792 | 0.005 |
| General function prediction only | 13.68339 | 11.49 | 2.64 | 0.051 | -52.678 | 0 |
| Inorganic ion transport and metabolism | 5.095706 | 5.05 | 1.16 | 0.023 | -1.844 | 0.065 |
| Intracellular trafficking, secretion, and vesicular transport | 0.724263 | 2.40 | 1.18 | 0.023 | 88.509 | 0 |
| Lipid transport and metabolism | 7.889291 | 3.23 | 1.41 | 0.028 | -127.508 | 0 |
| Nucleotide transport and metabolism | 2.224521 | 2.93 | 0.98 | 0.019 | 36.968 | 0 |
| Posttranslational modification, protein turnover, chaperones | 2.897051 | 3.90 | 1.17 | 0.023 | -52.548 | 0 |
| Replication, recombination and repair | 4.190378 | 6.19 | 2.23 | 0.046 | 49.533 | 0 |
| Signal transduction mechanisms | 4.138645 | 4.51 | 2.11 | 0.041 | 2.762 | 0.006 |
| Secondary metabolites biosynthesis, transport and catabolism | 5.431971 | 1.86 | 1.37 | 0.027 | -97.052 | 0 |
| Transcription | 9.932747 | 6.81 | 2.10 | 0.041 | -76.191 | 0 |
| Translation, ribosomal structure and biogenesis | 4.216244 | 7.39 | 4.03 | 0.079 | 55.110 | 0 |

^a^ Data from selected genomes in IMG bacterial genome database.

## Table S3 Genomic islands prediction by different methods

| **Start** | **End** | **Size** | **GI Prediction Program** |
| --- | --- | --- | --- |
| 955437 | 974728 | 19291 | Predicted by multiple methods |
| 1225784 | 1232799 | 7015 | Predicted by multiple methods |
| 1257307 | 1262114 | 4807 | Predicted by multiple methods |
| 2023735 | 2067073 | 43338 | Predicted by multiple methods |
| 2763649 | 2773795 | 10146 | Predicted by multiple methods |
| 3166243 | 3172232 | 5989 | Predicted by multiple methods |
| 3974497 | 3984935 | 10438 | Predicted by multiple methods |
| 2023735 | 2033529 | 9794 | SIGI-HMM |
| 3166243 | 3172232 | 5989 | SIGI-HMM |
| 955437 | 974728 | 19291 | IslandPath-DIMOB |
| 1225784 | 1232799 | 7015 | IslandPath-DIMOB |
| 1257307 | 1262114 | 4807 | IslandPath-DIMOB |
| 2023735 | 2067073 | 43338 | IslandPath-DIMOB |
| 2763649 | 2773795 | 10146 | IslandPath-DIMOB |
| 3974497 | 3984935 | 10438 | IslandPath-DIMOB |

## Table S4 Genes in GIs.

| **Locus Tag** | **From** | **To** | **Pro Length** | **Direction** | **Production** | Tax |
| --- | --- | --- | --- | --- | --- | --- |
| AS9A_0418 | 421124 | 421750 | 208 | + | putative Mce associated protein Mas1A | Rhodococcus equi 103S |
| AS9A_0419 | 422021 | 423028 | 335 | + | putative ABC transporter ATP-binding protein | Rhodococcus opacus B4 |
| AS9A_0420 | 423028 | 423837 | 269 | + | YrbE family protein | Rhodococcus erythropolis PR4 |
| AS9A_0424 | 427109 | 428104 | 331 | + | virulence factor Mce family protein | Rhodococcus equi ATCC 33707 |
| AS9A_0425 | 428104 | 429360 | 418 | + | putative Mce family protein Mce4D | Rhodococcus equi 103S |
| AS9A_0426 | 429432 | 430616 | 394 | + | putative Mce family protein | Nocardia farcinica IFM 10152 |
| AS9A_0427 | 430613 | 431863 | 416 | + | putative Mce family protein | Rhodococcus opacus B4 |
| AS9A_0429 | 432799 | 433503 | 234 | + | hypothetical membrane protein | Rhodococcus opacus B4 |
| AS9A_0430 | 434048 | 437371 | 1107 | + | DNA-directed RNA polymerase subunit beta | Nocardia farcinica IFM 10152 |
| AS9A_0431 | 437516 | 441469 | 1317 | + | DNA-directed RNA polymerase subunit beta prime | Rhodococcus equi ATCC 33707 |
| AS9A_0432 | 441603 | 442448 | 281 | - | hypothetical protein SCAB_53331 | Streptomyces scabiei 87.22 |
| AS9A_0433 | 442513 | 443259 | 248 | - | hypothetical protein RHA1_ro01953 | Rhodococcus jostii RHA1 |
| AS9A_0434 | 443276 | 443773 | 165 | - | hypothetical protein RHA1_ro01956 | Rhodococcus jostii RHA1 |
| AS9A_0626 | 661507 | 663096 | 529 | + | long-chain-fatty-acid--CoA ligase | Rhodococcus jostii RHA1 |
| AS9A_0811 | 841540 | 842196 | 218 | - | conserved hypothetical protein | Rhodococcus equi ATCC 33707 |
| AS9A_0812 | 842257 | 843261 | 334 | - | ABC superfamily ATP binding cassette transporter, ABC protein | Rhodococcus equi ATCC 33707 |
| AS9A_0813 | 843379 | 844149 | 256 | - | putative transcriptional regulator | Sorangium cellulosum 'So ce 56' |
| AS9A_0814 | 844290 | 845276 | 328 | + | daunorubicin resistance ABC superfamily ATP binding cassette transporter, ABC protein | Aeromicrobium marinum DSM 15272 |
| AS9A_0815 | 845276 | 846088 | 270 | + | ABC-2 type transporter | Cellulomonas flavigena DSM 20109 |
| AS9A_0816 | 846116 | 846721 | 201 | - | TetR family transcriptional regulator | Streptomyces avermitilis MA-4680 |
| AS9A_0817 | 846748 | 847704 | 318 | + | oxidoreductase | Saccharopolyspora erythraea NRRL 2338 |
| AS9A_1598 | 1660045 | 1661631 | 528 | + | FAD linked oxidase domain-containing protein | Nocardioides sp. JS614 |
| AS9A_1599 | 1661643 | 1662494 | 283 | + | diacylglycerol kinase, catalytic region | Nocardioides sp. JS614 |
| AS9A_1600 | 1662531 | 1663256 | 241 | - | ABC transporter permease | Rhodococcus jostii RHA1 |
| AS9A_1601 | 1663256 | 1664134 | 292 | - | putative ABC transporter ATP-binding protein | Rhodococcus opacus B4 |
| AS9A_1602 | 1664131 | 1664490 | 119 | - | putative transcriptional regulator | Nocardia farcinica IFM 10152 |
| AS9A_1603 | 1664677 | 1665252 | 191 | + | hypothetical protein ROP_66430 | Rhodococcus opacus B4 |
| AS9A_1604 | 1665239 | 1665901 | 220 | - | hypothetical protein RER_01490 | Rhodococcus erythropolis PR4 |
| AS9A_1605 | 1665898 | 1666620 | 240 | - | putative GntR family transcriptional regulator | Rhodococcus opacus B4 |
| AS9A_1606 | 1666707 | 1668083 | 458 | - | mycothione reductase | Rhodococcus erythropolis PR4 |
| AS9A_1607 | 1668124 | 1669200 | 358 | - | hypothetical protein RHA1_ro06611 | Rhodococcus jostii RHA1 |
| AS9A_1608 | 1669342 | 1670919 | 525 | + | malate dehydrogenase | Rhodococcus equi ATCC 33707 |
| AS9A_1609 | 1670974 | 1672914 | 646 | + | magnesium chelatase subunit | Rhodococcus erythropolis PR4 |
| AS9A_2993 | 3062090 | 3063526 | 478 | + | hypothetical protein pCLPp16 | Mycobacterium celatum |
| AS9A_2994 | 3063571 | 3064095 | 174 | - | MOSC domain-containing protein | Amycolatopsis mediterranei U32 |
| AS9A_3250 | 3333626 | 3333994 | 122 | - | ATP synthase epsilon chain | Rhodococcus opacus B4 |
| AS9A_3251 | 3334001 | 3335449 | 482 | - | ATP synthase F1 sector beta subunit | Rhodococcus equi ATCC 33707 |
| AS9A_3252 | 3335455 | 3336429 | 324 | - | ATP synthase F1 sector gamma subunit | Rhodococcus equi ATCC 33707 |
| AS9A_3253 | 3336478 | 3338118 | 546 | - | ATP synthase F1 sector alpha subunit | Rhodococcus equi ATCC 33707 |
| AS9A_3254 | 3338228 | 3339043 | 271 | - | F0F1 ATP synthase subunit delta | Rhodococcus jostii RHA1 |
| AS9A_3255 | 3339049 | 3339603 | 184 | - | ATP synthase subunit b | Rhodococcus opacus B4 |
| AS9A_3256 | 3339606 | 3339851 | 81 | - | ATP synthase subunit c | Rhodococcus erythropolis PR4 |
| AS9A_3257 | 3339977 | 3340864 | 295 | - | ATP synthase subunit a | Rhodococcus opacus B4 |
| AS9A_3258 | 3341072 | 3341554 | 160 | - | hypothetical protein RHA1_ro01479 | Rhodococcus jostii RHA1 |
| AS9A_3259 | 3341651 | 3342832 | 393 | - | glycosyl transferase family 4 | Rhodococcus equi 103S |
| AS9A_3260 | 3342838 | 3344145 | 435 | - | serine hydroxymethyltransferase | Rhodococcus erythropolis SK121 |
| AS9A_3261 | 3344166 | 3344828 | 220 | - | Sua5/YciO/YrdC/YwlC family protein | Gordonia bronchialis DSM 43247 |
| AS9A_3263 | 3344911 | 3345765 | 284 | - | HemK family methyltransferase | Rhodococcus jostii RHA1 |
| AS9A_3264 | 3345889 | 3346377 | 162 | + | 0 |  |
| AS9A_3267 | 3348355 | 3348585 | 76 | - | ribosomal protein L31 | Tsukamurella paurometabola DSM 20162 |
| AS9A_3268 | 3348737 | 3350836 | 699 | - | transcription termination factor Rho | Rhodococcus opacus B4 |
| AS9A_3269 | 3351101 | 3352078 | 325 | - | homoserine kinase | Rhodococcus equi ATCC 33707 |
| AS9A_3270 | 3352075 | 3353157 | 360 | - | threonine synthase | Rhodococcus jostii RHA1 |
| AS9A_3271 | 3353172 | 3354503 | 443 | - | homoserine dehydrogenase | Rhodococcus equi ATCC 33707 |
| AS9A_3533 | 3604247 | 3605482 | 411 | - | hypothetical protein CMM_0178 | Clavibacter michiganensis subsp. michiganensis NCPPB 382 |
| AS9A_3534 | 3605521 | 3606429 | 302 | - | putative Mn2+/Zn2+ ABC-type transporter,substrate binding protein | Clavibacter michiganensis subsp. michiganensis NCPPB 382 |
| AS9A_3535 | 3606426 | 3607709 | 427 | - | hypothetical protein CMM_0176 | Clavibacter michiganensis subsp. michiganensis NCPPB 382 |
| AS9A_3536 | 3607719 | 3608417 | 232 | - | ABC metal ion transporter, ATP-binding component | Rhodococcus jostii RHA1 |
| AS9A_3537 | 3608485 | 3609351 | 288 | + | putative Mn2+/Zn2+ ABC-type transporter,permease component | Clavibacter michiganensis subsp. michiganensis NCPPB 382 |
| AS9A_3538 | 3609358 | 3610302 | 314 | - | hypothetical protein MintA_01489 | Mycobacterium intracellulare ATCC 13950 |
| AS9A_3539 | 3610337 | 3610777 | 146 | - | ChaB family protein | Mycobacterium parascrofulaceum ATCC BAA-614 |
| AS9A_3540 | 3610994 | 3611275 | 93 | + | 0 |  |
| AS9A_3541 | 3611280 | 3614726 | 1148 | - | hypothetical protein REQ_16590 | Rhodococcus equi 103S |
| AS9A_3542 | 3615267 | 3616031 | 254 | + | ABC transporter ATP-binding protein | Rhodococcus erythropolis PR4 |
| AS9A_3567 | 3637751 | 3639157 | 468 | - | sensor histidine kinase | Rhodococcus equi ATCC 33707 |
| AS9A_3568 | 3639154 | 3639843 | 229 | - | two component transcriptional regulator | Mycobacterium vanbaalenii PYR-1 |
| AS9A_3569 | 3639910 | 3640518 | 202 | - | hypothetical protein RER_43990 | Rhodococcus erythropolis PR4 |
| AS9A_3570 | 3640608 | 3640784 | 58 | - | 50S ribosomal protein L32 | Salinispora tropica CNB-440 |
| AS9A_3571 | 3640798 | 3641073 | 91 | - | 50S ribosomal protein L31 | Corynebacterium kroppenstedtii DSM 44385 |
| AS9A_3572 | 3641073 | 3642341 | 422 | - | hypothetical protein ROP_56820 | Rhodococcus opacus B4 |
| AS9A_3573 | 3642455 | 3642571 | 38 | - | 0 |  |
| AS9A_4277 | 4413424 | 4413681 | 85 | + | unknown | Rhodococcus erythropolis |
| AS9A_4278 | 4413683 | 4413940 | 85 | + | toxin-antitoxin system, toxin component, Txe/YoeB family | Kytococcus sedentarius DSM 20547 |
| AS9A_4279 | 4414085 | 4414240 | 51 | + | 0 |  |
| AS9A_4280 | 4414196 | 4415401 | 401 | - | IS30 family transposase | Mycobacterium parascrofulaceum ATCC BAA-614 |
| AS9A_4281 | 4415442 | 4415684 | 80 | + | hypothetical protein AMED_8733 | Amycolatopsis mediterranei U32 |
| AS9A_4282 | 4416088 | 4416726 | 212 | - | TetR family transcriptional regulator | Mycobacterium parascrofulaceum ATCC BAA-614 |
| AS9A_4283 | 4417034 | 4417558 | 174 | + | thioesterase family protein | Rhodococcus erythropolis SK121 |
| AS9A_4285 | 4417711 | 4418712 | 333 | - | transcriptional regulatory protein | Rhodococcus erythropolis SK121 |
| AS9A_4286 | 4418831 | 4419151 | 106 | + | ferredoxin | Mycobacterium gilvum PYR-GCK |
| AS9A_4287 | 4419274 | 4420563 | 429 | + | cytochrome P450 | Rhodococcus erythropolis SK121 |
| AS9A_4288 | 4420560 | 4421786 | 408 | + | FAD-dependent pyridine nucleotide-disulphide oxidoreductase | Mycobacterium gilvum PYR-GCK |
| AS9A_4289 | 4422000 | 4423061 | 353 | + | conserved hypothetical protein | Rhodococcus erythropolis SK121 |
| AS9A_4290 | 4423236 | 4423775 | 179 | - | putative transcriptional regulator | Nocardia farcinica IFM 10152 |
| AS9A_4291 | 4423780 | 4425390 | 536 | + | putative transporter | Nocardia farcinica IFM 10152 |
| AS9A_4292 | 4425360 | 4426718 | 452 | - | transposase mutator type | Gordonia bronchialis DSM 43247 |
| AS9A_4294 | 4427383 | 4428066 | 227 | + | copper/silver-translocating P-type ATPase | Kytococcus sedentarius DSM 20547 |
| AS9A_4295 | 4428063 | 4428197 | 44 | + | copper-translocating P-type ATPase | Rhodococcus erythropolis SK121 |
| AS9A_4296 | 4428503 | 4428970 | 155 | - | 0 |  |
| AS9A_4297 | 4429161 | 4430357 | 398 | + | cytochrome P450 | Nocardioides sp. JS614 |
| AS9A_4298 | 4430636 | 4431223 | 195 | + | Resolvase domain protein | Arthrobacter chlorophenolicus A6 |
| AS9A_4299 | 4431861 | 4432763 | 300 | + | hypothetical protein CMS_2296 | Clavibacter michiganensis subsp. sepedonicus |
| AS9A_4300 | 4432912 | 4434675 | 587 | + | Eco57I restriction endonuclease | Shewanella loihica PV-4 |
| AS9A_4301 | 4434672 | 4435766 | 364 | + | BsuBIPstI restriction endonuclease domain-containing protein | Shewanella loihica PV-4 |
| AS9A_4302 | 4435926 | 4436633 | 235 | + | hypothetical protein Bfae_16700 | Brachybacterium faecium DSM 4810 |
| AS9A_4303 | 4436659 | 4436988 | 109 | - | ABC transporter related protein | Desulfarculus baarsii DSM 2075 |
| AS9A_4304 | 4437036 | 4437809 | 257 | - | 0 |  |
| AS9A_4305 | 4437953 | 4440700 | 915 | + | LAL subfamily transcriptional regulator | Streptomyces hygroscopicus ATCC 53653 |
| AS9A_4306 | 4440818 | 4441129 | 103 | + | hypothetical protein StAA4_36236 | Streptomyces sp. AA4 |
| AS9A_4308 | 4441289 | 4442581 | 430 | + | putative alcohol dehydrogenase | Nocardia farcinica IFM 10152 |
| AS9A_4309 | 4442652 | 4443788 | 378 | + | hypothetical protein nfa22250 | Nocardia farcinica IFM 10152 |
| AS9A_4310 | 4443796 | 4445292 | 498 | + | hypothetical protein nfa22260 | Nocardia farcinica IFM 10152 |
| AS9A_4311 | 4445292 | 4446206 | 304 | + | hypothetical protein nfa22270 | Nocardia farcinica IFM 10152 |
| AS9A_4312 | 4446275 | 4447123 | 282 | - | 3-hydroxyacyl-CoA dehydrogenase | Rhodococcus opacus B4 |
| AS9A_4313 | 4447250 | 4448410 | 386 | - | acyl-CoA dehydrogenase | Rhodococcus jostii RHA1 |
| AS9A_4314 | 4448500 | 4449237 | 245 | - | regulatory protein | Rhodococcus erythropolis SK121 |
| AS9A_4315 | 4449317 | 4450801 | 494 | - | NCS1 nucleoside transporter | Streptomyces hygroscopicus ATCC 53653 |
| AS9A_4316 | 4451016 | 4452467 | 483 | + | C4-dicarboxylate anaerobic carrier | Halomonas elongata DSM 2581 |
| AS9A_4317 | 4454252 | 4455997 | 581 | - | putative oxidoreductase | Streptomyces scabiei 87.22 |
| AS9A_4318 | 4456044 | 4457189 | 381 | + | long-chain-acyl-CoA dehydrogenase | Rhodococcus jostii RHA1 |
| AS9A_4319 | 4457173 | 4458201 | 342 | + | 2-nitropropane dioxygenase | Rhodococcus jostii RHA1 |

## Table S5 Comparison of the frequency of gene transfer events between DQS3-9A1^T^ and 118 other bacteria

|  | Number of GIs | | Percentage of the whole genome length | | |
| --- | --- | --- | --- | --- | --- |
| Methods | DQS3-9A1^T^ | Average | | DQS3-9A1^T^ | Average |
| IslandPath - DIMOB | 2 | 85 | | 0.32% | 5.3% |
| SGI-HMM | 6 | 10 | | 1.95% | 4.02 |

## Table S6 Genes discussed and described in this work

| **Locus Tag** | **From** | **To** | **Pro Length** | **Gene** | **Annotation Name** |
| --- | --- | --- | --- | --- | --- |
| **Gene encoding alkane hydroxylase** | | | | | |
| AS9A_2113 | 2193077 | 2194318 | 413 | *alkB* | alkane-1-monooxygenase |
| AS9A_2121 | 2205195 | 2206421 | 408 | *alkB* | alkane-1-monooxygenase |
| AS9A_3799 | 3874844 | 3876094 | 416 | *alkB* | alkane-1-monooxygenase |
| AS9A_2813 | 2854659 | 2866047 | 462 | *CYP153* | putative Linalool 8-monooxygenase |
| AS9A_4287 | 4419274 | 4420563 | 429 | *CYP153* | cytochrome P450 |
| AS9A_3890 | 3961355 | 3962767 | 470 | *ladA* | monooxygenase |
| AS9A_2156 | 2238884 | 2239225 | 113 | *prmD* | putative monooxygenase subunit |
| AS9A_2157 | 2239222 | 2240328 | 368 | *prmC* | monooxygenase hydroxylase |
| AS9A_2158 | 2240389 | 2241432 | 347 | *prmB* | putative phenol hydroxylase |
| AS9A_2159 | 2241519 | 2243150 | 543 | *prmA* | propane monooxygenase hydroxylase large subunit |
| **Genes involved in Na^+^ and K^+^ transport** | | | | | |
| AS9A_0226 | 239730 | 240086 | 118 | *mnhG* | monovalent cation/proton antiporter subunit MnhG/PhaG |
| AS9A_0227 | 240083 | 240343 | 86 | *mnhF* | pH adaptation potassium efflux system protein |
| AS9A_0228 | 240340 | 240747 | 135 | *mnhE* | Na+/H+ ion antiporter family protein |
| AS9A_0229 | 240744 | 242240 | 498 | *mnhD* | NADH/Ubiquinone/plastoquinone (Complex I) |
| AS9A_0230 | 242237 | 242704 | 155 | *mnhC* | NADH-ubiquinone oxidoreductase chain 4L |
| AS9A_0231 | 242701 | 245556 | 951 | *mnhA* | NADH dehydrogenase (Quinone) |
| AS9A_0556 | 590174 | 590506 | 110 | *mnhG* | multisubunit Na+/H+ antiporter subunit G |
| AS9A_0557 | 590503 | 590772 | 89 | *mnhF* | mnhF gene product |
| AS9A_0558 | 590778 | 591329 | 183 | *mnhE* | putative multisubunit Na+:H+ antiporter MnhE subunit |
| AS9A_0559 | 591326 | 592975 | 549 | *mnhD* | mnhD gene product |
| AS9A_0560 | 592975 | 593487 | 170 | *mnhC* | mnhC gene product |
| AS9A_0561 | 593484 | 596453 | 989 | *mnhA* | NADH-Ubiquinone/plastoquinone |
| AS9A_1548 | 1610985 | 1611233 | 82 | *mnhF* | hypothetical protein |
| AS9A_1549 | 1611230 | 1611544 | 104 | *mnhG* | Na+/H+ antiporter subunit |
| AS9A_1550 | 1611541 | 1612467 | 308 | *mnhB* | hypothetical protein |
| AS9A_1551 | 1612464 | 1612772 | 102 | *mnhC* | hypothetical protein |
| AS9A_1552 | 1612765 | 1614267 | 500 | *mnhD* | fomrate hydrogenlyase subunit 3/multisubunit Na+/H+ antiporter subunit MnhD |
| AS9A_1553 | 1614269 | 1615777 | 502 | *mnhD* | fomrate hydrogenlyase subunit 3/multisubunit Na+/H+ antiporter subunit MnhD |
| AS9A_1554 | 1615777 | 1617552 | 591 | *mnhD* | fomrate hydrogenlyase subunit 3/multisubunit Na+/H+ antiporter subunit MnhD |
| AS9A_1555 | 1617549 | 1618064 | 171 | *mnhE* | multisubunit Na+/H+ antiporter subunit MnhE |
| AS9A_0248 | 262317 | 262991 | 224 | *trkA* | trkA gene product |
| AS9A_0249 | 262981 | 264357 | 458 | *trkH* | putative cation transporter |
| AS9A_4251 | 4381422 | 4382762 | 446 | *trkH* | putative cation transporter |
| AS9A_4252 | 4382971 | 4383825 | 284 | *trkA* | transporter, cation channel family |
| AS9A_2378 | 2438514 | 2440313 | 599 | *nhaC* | *nhaC* gene product |
| AS9A_2640 | 2682968 | 2684227 | 419 | *nhaC* | sodium/hydrogen exchanger |
| AS9A_3483 | 3554295 | 3555542 | 415 | *nhaC* | sodium/hydrogen exchanger |
| AS9A_4549 | 4696485 | 4698476 | 663 | *nhaC* | sodium/hydrogen exchanger |
| **Other genes described and discussed in this work** | | | | | |
| AS9A_0979 | 1005429 | 1007180 | 583 | *betP* | choline/carnitine/betaine transport |
| AS9A_1011 | 1042038 | 1043945 | 635 | *betP* | glycine betaine transport integral membrane protein BetP |
| AS9A_2468 | 2523211 | 2525334 | 707 | *betP* | choline/carnitine/betaine transporter |
| AS9A_2953 | 3016122 | 3017732 | 536 | *betP* | choline/carnitine/betaine transporter |
| AS9A_3514 | 3586989 | 3588962 | 657 | *betP* | glycine betaine transport integral membrane protein BetP |
| AS9A_1074 | 1108204 | 1109682 | 492 |  | Na^+^/solute symporter |
| AS9A_2078 | 2152297 | 2152980 | 227 |  | ABC transporter permease |
| AS9A_2079 | 2152977 | 2153708 | 243 |  | binding-protein-dependent transport systems inner membrane component |
| AS9A_2080 | 2153705 | 2154823 | 372 |  | ABC transporter ATP-binding protein |
| AS9A_2081 | 2154823 | 2155725 | 300 |  | Substrate-binding region of ABC-type glycine betaine transport system |
| AS9A_2085 | 2159365 | 2160156 | 263 |  | ABC amino acid transporter, ATPase component |
| AS9A_2086 | 2160137 | 2160784 | 215 |  | ABC amino acid transporter, permease component |
| AS9A_2087 | 2160787 | 2161515 | 242 |  | putative ABC transporter permease protein |
| AS9A_2088 | 2161512 | 2162372 | 286 |  | ABC amino acid transporter, extracellular solute binding component |
| AS9A_3012 | 3085435 | 3086202 | 255 |  | ectoine/hydroxyectoine ABC transporter, ATP-binding protein |
| AS9A_2789 | 2840359 | 2841915 | 518 | *putP* | sodium/proline symporter |
| AS9A_3791 | 3867693 | 3869399 | 568 | *mtrB* | sensor histidine kinase MtrB |
| AS9A_3792 | 3869450 | 3870133 | 227 | *mtrA* | DNA-binding response regulator MtrA |
| AS9A_4249 | 4379299 | 4379979 | 226 | *kdpE* | KDP operon transcriptional regulatory protein KdpE |
| AS9A_4250 | 4379976 | 4381325 | 449 | *kdpD* | osmosensitive K channel His kinase sensor |
| AS9A_0138 | 146543 | 154168 | 2541 |  | putative non-ribosomal peptide synthetase |
| AS9A_0140 | 155316 | 159782 | 1488 |  | putative non-ribosomal peptide synthetase |
| AS9A_0303 | 316847 | 320809 | 1320 |  | non-ribosomal peptide synthetase |
| AS9A_0454 | 464595 | 484877 | 6760 |  | non-ribosomal peptide synthetase |
| AS9A_2979 | 3048088 | 3049437 | 449 |  | putative non-ribosomal peptide synthetase |
| AS9A_3751 | 3825126 | 3826916 | 596 |  | putative non-ribosomal peptide synthetase |
| AS9A_4071 | 4141859 | 4164025 | 7388 |  | non-ribosomal peptide synthetase |
| AS9A_4425 | 4553351 | 4575796 | 7481 |  | non-ribosomal peptide synthetase |
| AS9A_0147 | 166459 | 167340 | 293 |  | putative glycosyltransferase |
| AS9A_0623 | 658606 | 660003 | 465 |  | glycosyltransferase |
| AS9A_0687 | 720428 | 721651 | 407 |  | putative glycosyltransferase |
| AS9A_0794 | 823173 | 824534 | 453 |  | putative glycosyltransferase |
| AS9A_1402 | 1460340 | 1461704 | 454 |  | putative glycosyltransferase |
| AS9A_1699 | 1765001 | 1766200 | 399 |  | glycosyltransferase |
| AS9A_1608 | 1669342 | 1670919 | 525 | *mqo* | malate: quinine oxidoreductase |
| AS9A_3917 | 3984682 | 3985083 | 133 | *sdhC* | succinate dehydrogenase cytochrome b subunit |
| AS9A_3918 | 3985197 | 3985538 | 113 | *sdhD* | succinate dehydrogenase hydrophobic membrane anchor protein |
| AS9A_3919 | 3985550 | 3987316 | 588 | *sdhA* | succinate dehydrogenase flavoprotein subunit |
| AS9A_3920 | 3987316 | 3988092 | 258 | *sdhB* | succinate dehydrogenase |
| AS9A_0231 | 242701 | 245556 | 951 | *ndh* | NADH dehydrogenase |
| AS9A_2465 | 2520432 | 2521805 | 457 | *ndh* | NADH dehydrogenase |
| AS9A_3118 | 3186709 | 3188112 | 467 | *ndh* | NADH dehydrogenase |
| AS9A_2092 | 2168743 | 2172447 | 1234 | *narG* | putative nitrate reductase alpha subunit |
| AS9A_2093 | 2172447 | 2174087 | 546 | *narH* | putative nitrate reductase beta subunit |
| AS9A_2094 | 2174084 | 2174788 | 234 | *narJ* | putative nitrate reductase delta subunit |
| AS9A_2095 | 2174785 | 2175516 | 243 | *narI* | respiratory nitrate reductase gamma subunit |
| AS9A_0114 | 123608 | 123913 | 101 | *nasE* | nitrite reductase [NAD(P)H] small subunit [Rhodococcus jostii RHA1] |
| AS9A_0115 | 123910 | 126390 | 826 | *nasD* | nitrite reductase [NAD(P)H] large subunit [Rhodococcus jostii RHA1] |
| AS9A_3757 | 3830523 | 3830912 | 129 |  | nitrite reductase (NAD(P)H), small subunit] |
| AS9A_3758 | 3830912 | 3833443 | 843 |  | nitrite reductase (NAD(P)H)large subunit |
| AS9A_2041 | 2111976 | 2113154 | 392 | *nirA* | ferredoxin-nitrite reductase |

## Table S7 Genes involved in TCS

| **Locus Tag** | **Product Name** | **Func ID** | **Func Name** |
| --- | --- | --- | --- |
| AS9A_0127 | sensor kinase, two-component system |  |  |
| AS9A_0128 | putative NarL family two-component response regulator | K02479 | two-component system, NarL family, response regulator |
| AS9A_2055 | Receptor-like histidine kinase of 2 component system |  |  |
| AS9A_2056 | putative NarL family two-component response regulator | K02479 | two-component system, NarL family, response regulator |
| AS9A_2312 | sensor kinase, two-component system | K02480 | two-component system, NarL family, sensor kinase [EC:2.7.13.3] |
| AS9A_2313 | LuxR family DNA-binding response regulator | K07684 | two-component system, NarL family, nitrate/nitrite response regulator NarL |
| AS9A_2364 | putative two-component system sensor kinase | K02484 | two-component system, OmpR family, sensor kinase [EC:2.7.13.3] |
| AS9A_2365 | putative OmpR family two-component response regulator | K02483 | two-component system, OmpR family, response regulator |
| AS9A_3081 | two component LuxR familytranscriptional regulator | K11618 | two-component system, NarL family, response regulator LiaR |
| AS9A_3082 | Two-component sensor kinase |  |  |
| AS9A_3567 | sensor histidine kinase | K07653 | two-component system, OmpR family, sensor histidine kinase MprB [EC:2.7.13.3] |
| AS9A_3568 | response regulator mprA | K07669 | two-component system, OmpR family, response regulator MprA |
| AS9A_3773 | transcription factor WhiB |  |  |
| AS9A_3774 | putative two-component histidine kinase | K00936 |  |
| AS9A_3791 | mtrB gene product | K07654 | two-component system, OmpR family, sensor histidine kinase MtrB [EC:2.7.13.3] |
| AS9A_3792 | DNA-binding response regulator MtrA | K07670 | two-component system, OmpR family, response regulator MtrA |
| AS9A_3822 | two component system response regulator | K02479 | two-component system, NarL family, response regulator |
| AS9A_3823 | putative two-component histidine kinase | K07673 | two-component system, NarL family, nitrate/nitrite sensor histidine kinase NarX [EC:2.7.13.3] |
| AS9A_3828 | sensor kinase, two-component system |  |  |
| AS9A_3829 | response regulator, two-component system | K07657 | two-component system, OmpR family, phosphate regulon response regulator PhoB |
| AS9A_3906 | Histidine kinase | K07778 | two-component system, NarL family, sensor histidine kinase DesK [EC:2.7.13.3] |
| AS9A_3907 | Two component LuxR family transcriptional regulator | K02479 | two-component system, NarL family, response regulator |
| AS9A_4020 | putative two-component system response regulator | K11618 | two-component system, NarL family, response regulator LiaR |
| AS9A_4021 | sensor histidine kinase |  |  |
| AS9A_4249 | phosphate regulon transcriptional regulator PhoB | K07667 | two-component system, OmpR family, KDP operon response regulator KdpE |
| AS9A_4250 | osmosensitive K channel His kinase sensor | K07646 | two-component system, OmpR family, sensor histidine kinase KdpD [EC:2.7.13.3] |
| AS9A_4323 | putative two-component system response regulator | K02479 | two-component system, NarL family, response regulator |
| AS9A_4324 | putative two-component system sensor kinase | K11617 | two-component system, NarL family, sensor histidine kinase LiaS [EC:2.7.13.3] |
| AS9A_4484 | two component system histidine kinase | K07701 | two-component system, CitB family, sensor histidine kinase DcuS [EC:2.7.13.3] |
| AS9A_4485 | response regulator of citrate/malate metabolism | K11692 | two-component system, CitB family, response regulator DctR |
| AS9A_P20012 | Two component transcriptional regulator | K07684 | two-component system, NarL family, nitrate/nitrite response regulator NarL |
| AS9A_P20013 | putative two-component histidine kinase | K07675 | two-component system, NarL family, sensor histidine kinase UhpB [EC:2.7.13.3] |

## Table S8 Genes in compatible solutes transport and biosynthesis

| **Locus Tag** | **From** | **To** | **Pro Length** | **Direction** | **Product Name** |
| --- | --- | --- | --- | --- | --- |
| AS9A_0791 | 820251 | 821705 | 484 | - | betaine-aldehyde dehydrogenase |
| AS9A_0979 | 1005429 | 1007180 | 583 | + | choline/carnitine/betaine transport |
| AS9A_1011 | 1042038 | 1043945 | 635 | + | glycine betaine transport integral membrane protein BetP |
| AS9A_1012 | 1044039 | 1045628 | 529 | - | glucose-methanol-choline oxidoreductase |
| AS9A_1074 | 1108204 | 1109682 | 492 | - | Na^+^/solute symporter |
| AS9A_1131 | 1175051 | 1175446 | 131 | - | ectoine synthase |
| AS9A_1157 | 1201780 | 1203411 | 543 | - | glucose-methanol-choline oxidoreductase |
| AS9A_1664 | 1726727 | 1730194 | 1155 | - | bifunctional proline dehydrogenase/pyrroline-5-carboxylate dehydrogenase |
| AS9A_1666 | 1731384 | 1731758 | 124 | + | sodium/proline symporter |
| AS9A_2077 | 2151333 | 2152253 | 306 | + | ectoine hydroxylase |
| AS9A_2078 | 2152297 | 2152980 | 227 | - | ABC transporter permease |
| AS9A_2079 | 2152977 | 2153708 | 243 | - | binding-protein-dependent transport systems inner membrane component |
| AS9A_2080 | 2153705 | 2154823 | 372 | - | ABC transporter ATP-binding protein |
| AS9A_2081 | 2154823 | 2155725 | 300 | - | Substrate-binding region of ABC-type glycine betaine transport system |
| AS9A_2085 | 2159365 | 2160156 | 263 | - | ABC amino acid transporter, ATPase component |
| AS9A_2086 | 2160137 | 2160784 | 215 | - | ABC amino acid transporter, permease component |
| AS9A_2087 | 2160787 | 2161515 | 242 | - | putative ABC transporter permease protein |
| AS9A_2088 | 2161512 | 2162372 | 286 | - | ABC amino acid transporter, extracellular solute binding component |
| AS9A_2468 | 2523211 | 2525334 | 707 | - | choline/carnitine/betaine transporter |
| AS9A_2469 | 2525340 | 2526998 | 552 | - | choline dehydrogenase |
| AS9A_2701 | 2745999 | 2747582 | 527 | - | choline dehydrogenase |
| AS9A_2789 | 2840359 | 2841915 | 518 | + | sodium/proline symporter |
| AS9A_2953 | 3016122 | 3017732 | 536 | + | choline/carnitine/betaine transporter |
| AS9A_3012 | 3085435 | 3086202 | 255 | + | ectoine/hydroxyectoine ABC transporter, ATP-binding protein |
| AS9A_3514 | 3586989 | 3588962 | 657 | + | glycine betaine transport integral membrane protein BetP |
| AS9A_4539 | 4686514 | 4687437 | 307 | + | Proline dehydrogenase |
